# Integrative Bioinformatic Approach for microRNA Interactome Networks in Human Papillomavirus-16 Infection

**DOI:** 10.5152/eurasianjmed.2025.25817

**Published:** 2025-09-30

**Authors:** Berkcan Doğan

**Affiliations:** 1 Department of Medical Genetics, ursa Uludağ University Faculty of Medicine, Bursa, Türkiye; 2 Department of Translational Medicine, Bursa Uludağ University Institute of Health Sciences, Bursa, Türkiye

**Keywords:** Human papillomavirus-16, microRNA, interactome, host–virus interactions, bioinformatic analysis, cancer datasets

## Abstract

**Objective:**

High-risk human papillomavirus (HPV), particularly HPV-16, is a major driver of carcinogenesis. Despite advances in understanding HPV-mediated oncogenesis, the role of microRNA (miRNA) interactome networks in HPV-16-driven tumorigenesis remains unclear. Using an integrative bioinformatic approach, this study identified key miRNAs, target genes, and transcription factors (TFs) involved in HPV-16–associated cancers.

**Methods:**

Human papillomavirus-16–associated miRNAs were retrieved from viRBase. microRNAs and their interactors were analyzed using The Cancer Genome Atlas and Genotype-tissue Expression datasets to investigate the expression patterns and potential roles in carcinogenesis. microRNA–messenger RNA (mRNA) interactions, TFs enrichment, Kyoto Encyclopedia of Genes and Genomes (KEGG) pathways, and GO terms analyses uncovered molecular networks disrupted by HPV-16. Receiver operating characteristic curve (ROC) and Kaplan-Meier analyses assessed the clinical significance of dysregulated miRNAs.

**Results:**

Eight miRNAs (hsa-miR-16-5p, hsa-miR-24-3p, hsa-miR-34a-5p, hsa-miR-100-5p, hsa-miR-125b-5p, hsa-miR-203a-3p, hsa-miR-205-5p, and hsa-miR-331-3p) were significantly dysregulated in HPV-16 infection and enriched in key KEGG pathways, highlighting involvement in cellular processes and regulatory mechanisms. Among these, hsa-miR-100-5p, hsa-miR-125b-5p, and hsa-miR-331-3p were the most significant in HPV-16-driven cancer types, with hsa-miR-125b-5p emerging as a key prognostic regulator. *MAP3K13* and *NR1H4* were identified as critical gene and TF candidates in HPV-16 carcinogenesis.

**Conclusion:**

This study provides novel insights into miRNA interactome networks in HPV-16–driven carcinogenesis, identifying biomarkers and therapeutic targets. Integrating translational bioinformatic insights with experimental validation paves the way for developing targeted diagnostic and therapeutic strategies and unravelling complex host–virus interactions, ultimately enhancing the management of HPV-associated cancers.

**Main Points**

- hsa-miR-100-5p, hsa-miR-125b-5p, and hsa-miR-331-3p were the most significantly altered miRNAs, while breast cancer (BRCA) and lung squamous cell carcinoma (LUSC) exhibited the highest number of dysregulated miRNAs.
- Receiver operating characteristic curve and Kaplan-Meier analyses showed strong diagnostic and prognostic potential of hsa-miR-125b-5p in TCGA datasets.
- *CLOCK* and *MGAT4A* were found to interact with 2 distinct KEGG pathways, suggesting their involvement in HPV-16–related oncogenesis.
- *NR1H4* was identified as a key TF associated with hsa-miR-100-5p and hsa-miR-34a-5p, indicating its potential role in miRNA-mediated regulation.

## Introduction

Approximately 5% of all human cancers are caused by high-risk human papillomavirus (HPV) subtypes, including HPV-16, HPV-18, HPV-31, and HPV-45, which are responsible for cervical and other anogenital cancers.^1,2^ Among these, HPV-16 is the most prevalent subtype, accounting for 50-55% of all cases.^3,4^ The HPV-16 infection alters host cellular processes, leading to the dysregulation of key molecular pathways and promoting carcinogenesis.^5^ The viral oncoproteins E6 and E7 play a pivotal role in tumorigenesis by promoting the degradation of the tumor suppressor protein p53 and inactivating the retinoblastoma (Rb) family proteins, respectively, thereby impairing cell cycle regulation.^6,7^ The expression of E6 and E7 is typically enhanced after the integration of viral DNA into the host genome, where they interact with multiple cellular proteins and contribute to various cancer hallmarks.^8,9^ Additionally, E6 and E7 alter host microRNA (miRNA) expression,^10,11^ yet limited research has investigated the evaluation of miRNAs to HPV-derived carcinogenesis.^11-13^

MicroRNAs (miRNAs), short non-coding regulatory RNAs that control gene expression at the post-transcriptional level, have evolved as pivotal players in viral infections and cancer biology.^14,15^ microRNAs are master regulators of host–virus interactions, capable of directly interacting with viral genomes and modulating the host immune response.^16^ Human papillomaviruses, including HPV-16, can alter miRNA expression in infected cells, disrupting host defense mechanisms and establishing an environment that supports viral persistence and infection.^7,17^ However, the relationship between HPV-16 infection and the host miRNA interactome is still not fully understood.

This study aims to employ an integrative bioinformatic approach to unravel the miRNA interactome networks in HPV-16 infection. By integrating multi-omics data and advanced bioinformatic tools, this study provides novel insights into the miRNA interactome networks in HPV-16-derived carcinogenesis and potential biomarkers and therapeutic targets for HPV-associated cancers.

## Material and Methods

### microRNA Interactors in Human Papillomavirus-16 Infection

To identify miRNA-based interactomes associated with HPV-16, the VirBase (v.3.0) database was used, a comprehensive resource for the virus-host interactions platform.^18^ Employing the “Exact Search” option and applying the “virus–host interaction” filter, all interactions within the *Homo sapiens* were retrieved. As this study was conducted entirely using publicly available in-silico data, no ethical approval was required.

A score-based filtering approach was applied for downstream analyses using confidence scores (*S*) provided by VirBase. These scores are computed based on both the type and number of supporting evidence (experimental or predicted) using a logistic-weighted probabilistic model:



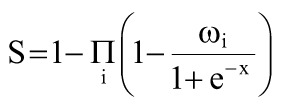



In this formula, *i *refers to evidence type (strong experimental evidence, weak experimental evidence, computational prediction method), *x* is the number of supporting resources for each type, and* w_i_* is the weight assigned to each evidence category (1.0 for strong experimental, 0.65 for weak experimental, and 0.25 for predictions). Interactions were stratified based on their confidence scores as follows:

- *S*≥0.7: were classified as high-confidence and included in core analyses,
- 0.4≤*S*<0.7: used in exploratory analyses,
- *S*<0.4: excluded to minimize false positives, as these are supported only by computational predictions.^18^

This scoring-based filtering enabled the prioritization of biologically relevant and experimentally validated host miRNA–virus interactions, thereby enhancing the robustness, accuracy, and interpretability of the downstream bioinformatic analyses.

### Pathway Enrichment Analyses

The identified miRNAs were functionally annotated using DIANA-miRPath (v.4.0), which integrates target-based functional analysis of miRNA with Kyoto Encyclopedia of Genes and Genomes (KEGG) and Gene Ontology (GO) analyses.^19^ The top 10 statistically significant KEGG pathways and GO:BP, GO:MF, and GO:CC terms were identified.

Pathway enrichment analysis was performed to identify significant molecular pathways and biological processes associated with HPV-16 infection. A significance threshold of *P* < .05 was applied to identify statistically enriched pathways. The pathway’s union merging method was applied exclusively to pathways with at least 2 interacting miRNAs. For heatmap visualization, adjusted *P*-values (FDR) were transformed into |–log_10_FDR| values to indicate enrichment strength in pathway clustering analyses.

### Differentially Expressed microRNAs in The Cancer Genome Atlas Datasets

The expression profiles of each miRNA across The Cancer Genome Atlas (TCGA) datasets were assessed using the CancerMIRNome database with the Wilcoxon Rank-Sum test to validate the significance of the determined miRNAs. CancerMIRNome is a comprehensive database integrating miRNome profiles from 33 TCGA cancer types using log_2_-transformed CPM (counts per million) values.^20^ Additionally, Receiver operating characteristic (ROC) analysis was performed to evaluate the diagnostic potential of the miRNAs. The ROC analysis was conducted with a *P-*value threshold of <.005 and an area under the curve (AUC) threshold of ≥0.90 to identify miRNAs with high predictive accuracy.

Kaplan-Meier survival analysis was performed using the CancerMIRNome database to evaluate the prognostic potential of these miRNAs. A high significance threshold of *P *< .005 was applied to determine miRNAs associated with survival outcomes.

### Target Genes and Transcription Factors of microRNA Interactors

The target genes of the identified interactors miRNAs were predicted using miRDB, a database that identifies target genes based on machine learning methods using high-throughput sequencing data.^21^ The target score threshold of ≤90 was applied to ensure high-confidence predictions.

The potential transcription factors (TFs) regulating these miRNAs were also predicted using TransmiR (v.3.0), a database that integrates experimentally validated miRNA–TF interactions.^22^ The association between the predicted target genes and their related miRNAs was statistically significant, with *P* < ..005.

### Pathways Analysis and Expression Patterns of microRNA Interactors

Pathway analysis of target genes and TFs was performed using Enrichr, a comprehensive gene set enrichment tool for pathway enrichment analysis.^23,24^ A significance threshold of *P* < ..05 was used for KEGG pathways.

Target genes and TFs of miRNAs were analyzed using RNA sequencing data from TCGA and the Genotype-Tissue Expression (GTEx) projects through Gene Expression Profiling Interactive Analysis 2 (GEPIA2).^25^ Each target gene and TF was evaluated separately within individual cancer datasets using analysis of variance. Genes and TFs with a *P* < .005 and |logFC|≥2 were identified as significantly differentially expressed.

## Results

### Determination of microRNA Interactors in Human Papillomavirus-16 Infection

The authors’ analysis identified a comprehensive network of miRNA interactomes associated with HPV-16 infection. A total of 11 distinct host-virus interactions involving HPV-16 were identified, focusing on viral proteins E6, E7, and L2 capsid (Table 1).^11-13,26-28^ Eight unique miRNAs were detected in various cell types, generally human foreskin keratinocytes (HFKs), human vaginal keratinocytes (HVKs), and different cancer types cell lines. Three of these miRNAs (hsa-miR-16-5p, hsa-miR-24-3p, and hsa-miR-203a-3p) showed in multiple interactions due to recurring associations. All miRNAs identified in HPV-16 interactions (Table 1) were included in downstream analyses without further selection. Interaction scores ranged from 0.7311 to 0.9933, with higher scores indicating stronger confidence in the interactions. The upregulated miRNAs, hsa-miR-16-5p, hsa-miR-24-3p, and hsa-miR-203a-3p, interacted with HPV-16 E6 and E7 oncoproteins. The hsa-miR-125b-5p was the only miRNA associated with HPV L2 capsid. E6 and E7 proteins were consistently involved in host–virus interactions, suggesting their critical roles in HPV-16 infection. The L2 capsid protein also showed interactions, indicating its potential role in viral entry or assembly.

### Pathway Enrichment Analysis of microRNA Interactors

**Figure 1.**
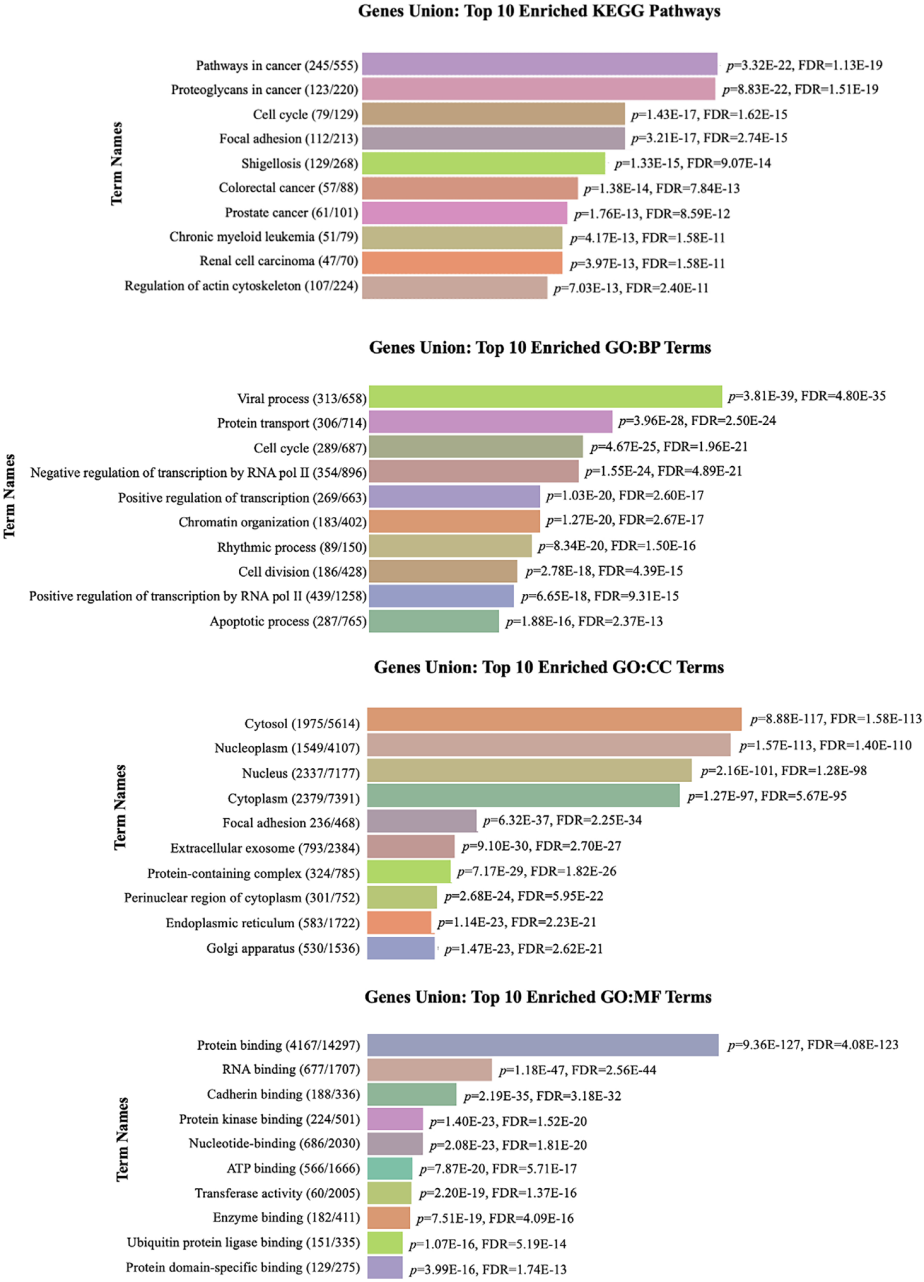
Top 10 significantly enriched KEGG pathways and GO terms for HPV-16-associated miRNA target genes (*P* < .05). Kyoto Encyclopedia of Genes and Genome pathways and GO terms are ranked by adjusted *P*-value (FDR). Each bar represents the pathway/term name, total number of genes involved, and number of genes hit in the authors’ data; BP, biological process; CC, cellular component; FDR, false discovery rate; GO, gene ontology; KEGG, Kyoto Encyclopedia of Genes and Genome; MF, molecular function.

Pathway enrichment analysis was performed using the KEGG pathway and GO functional analyses (Figure 1) to explore the functions of miRNA interactors in depth. The top-enriched KEGG pathways were involved in pathways in cancer, proteoglycans in cancer, cell cycle regulation, and various cancer types. Gene ontology analysis further revealed significant enrichment in key biological processes, with “viral process” being the most significantly enriched term (*P *< .05). Additionally, molecular functions, such as protein and RNA binding and protein kinase binding, were prominently enriched, underscoring the regulatory roles of miRNAs in HPV-16-associated molecular mechanisms.

**Figure 2.**
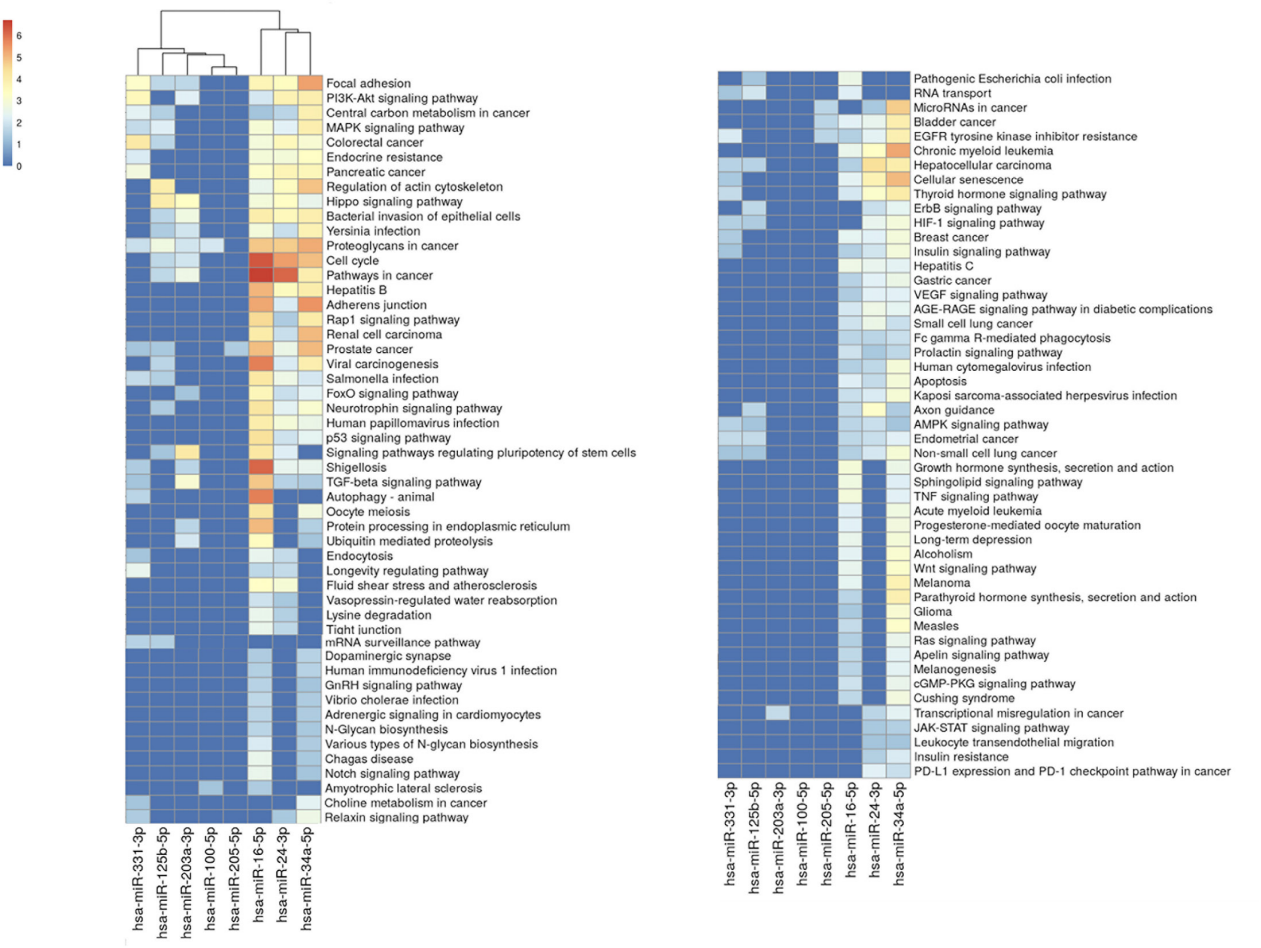
Functional clustering of significantly enriched pathways for interactor miRNAs in HPV-16. The heatmap displays pathways (rows) clustered by functional similarity (Euclidean distance) based on miRNA target gene overlap. Pathways were determined with Fisher’s exact test, and statistical significance was defined as *P* < .05. Color intensity represents enrichment strength |–log_10_FDR|, with red indicating the highest significance. FDR, false discovery rate; HPV, human papillomavirus; KEGG, Kyoto Encyclopedia of Genes and Genomes; miRNA, micro RNA.

The pathway enrichment analysis identified proteoglycans in cancer, pathways in cancer, and cell cycle regulation as the most significant pathways among 151 analyzed pathways (*P* < .05) (Figure 2, Supplementary Table 1). These findings highlight that almost all the identified miRNAs, particularly enriched in the “proteoglycans in cancer pathway,” include hsa-miR-16-5p, hsa-miR-24-3p, hsa-miR-34a-5p, hsa-miR-100-5p, hsa-miR-125b-5p, hsa-miR-203a-3p, and hsa-miR-331-3p. Moreover, “pathways in cancer” and “cell cycle regulation” pathways shared common 5 miRNA associations, both involving hsa-miR-16-5p, hsa-miR-24-3p, hsa-miR-34a-5p, hsa-miR-203a-3p, and hsa-miR-125b-5p (Figure 2). These findings highlight that multiple miRNAs collectively target host key molecular pathways, potentially involved in HPV-16 pathogenesis and subsequent carcinogenesis. Supplementary Table 1 includes detailed data for each miRNA and related pathway interactions, such as merged *P*-values and FDR values.

### Assessment of microRNAs Expression in The Cancer Genome Atlas Datasets

**Figure 3.**
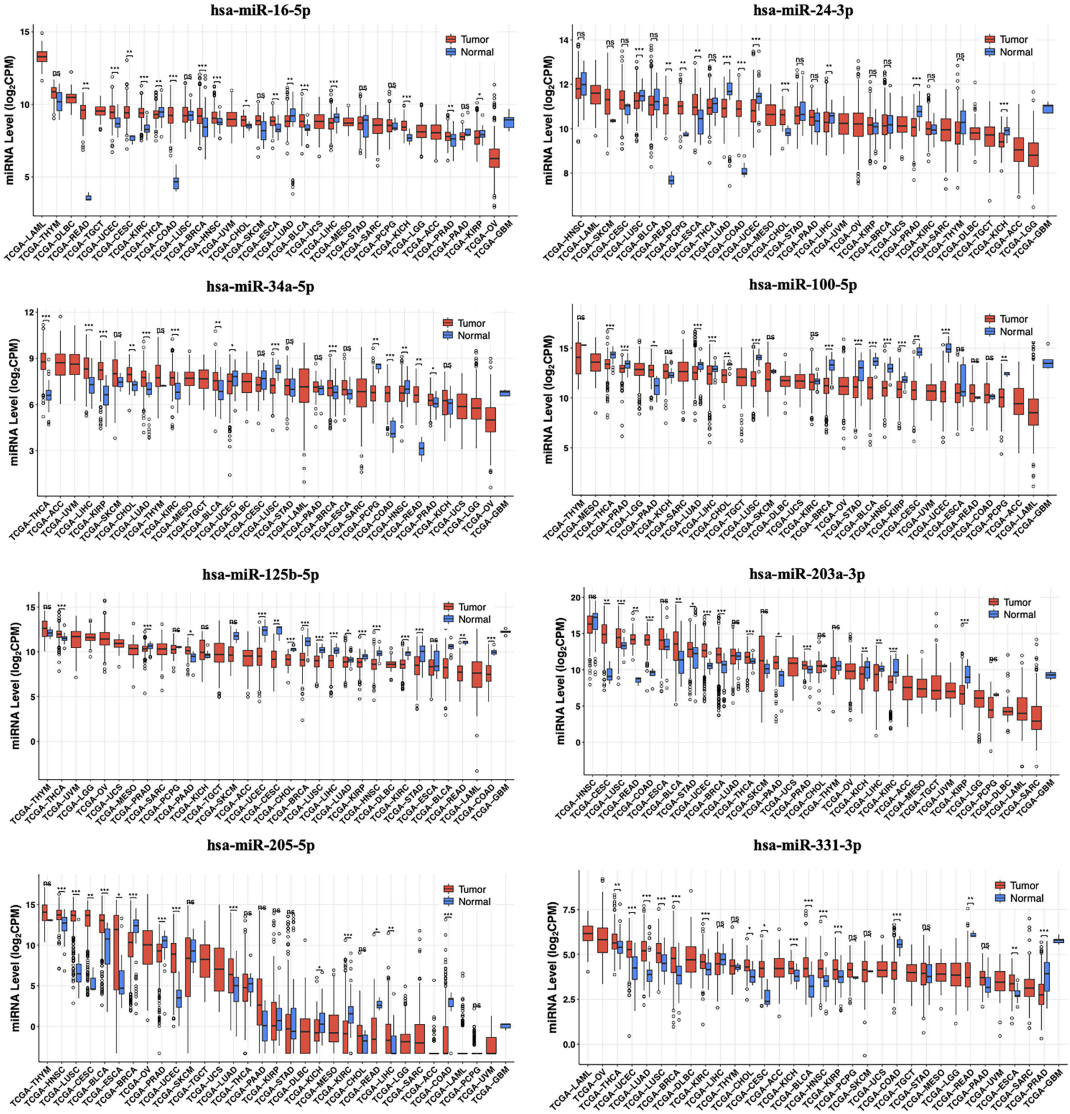
Differential expression of HPV-16-associated miRNAs in tumor vs. normal samples across he Cancer Genome Atlas (TCGA) datasets in 33 cancer types. Boxplots illustrate the expression levels of 8 miRNA interactors using log_2_-transformed CPM values. Blue boxes represent normal, and red boxes represent tumor samples. Statistical significance is indicated as follows: ^***^P < .001; ^**^P < .01; ^*^P < .05; ns (not significant): *P *> .05. CPM, counts per million; HPV, human papillomavirus; TCGA, The Cancer Genome Atlas.

The authors’ analysis revealed significant expression variations of interactor miRNAs across various cancer types (Figure 3). At least 2 miRNAs were significantly dysregulated (*P* < .001) in 15 out of the 33 analyzed cancer types. The most significant miRNAs were hsa-miR-100-5p, hsa-miR-125b-5p and hsa-miR-331-3p (n= 11), while breast cancer (BRCA) and lung squamous cell carcinoma (LUSC) showed the highest number of significant miRNA alterations (n= 7). The significantly dysregulated miRNAs in cancer datasets and the number of interactions were summarized in Supplementary Figure 1.

All miRNAs exhibited high diagnostic potential in ROC analysis, highlighting their utility as biomarkers in various cancer types (Table 2). The hsa-miR-125b-5p showed significant results across most cancer datasets, while cervical and endocervical cancers (CESC), colon adenocarcinoma (COAD), and rectum adenocarcinoma (READ) displayed the highest number of dysregulated miRNA interactions (AUC ≥ 0.90).

Kaplan-Meier survival analysis identified specific miRNAs significantly associated with patient prognosis (*P *< .005), with hsa-miR-125b-5p related to most cancer datasets and uveal melanoma (UVM) showing the highest number of prognostic miRNAs, which were hsa-miR-24-3p, hsa-miR-125b-5p, hsa-miR-203a-3p (n = 3) (Table 3). The hsa-miR-205-5p and hsa-miR-331-3p were not significant in any cancer dataset.

### Identification of Target Genes and TFs for microRNA Interactors

A total of 935 target genes (score ≥ 90) were identified as being associated with the miRNA interactors. Among these, the* MAP3K13* (Mitogen-Activated Protein Kinase 13) was targeted by 4 miRNAs (hsa-miR-16-5p, hsa-miR-125b-5p, hsa-miR-203a-3p, and hsa-miR-205-5p) while *CLOCK* (Clock Circadian Regulator), *MEX3C* (Mex-3 RNA Binding Family Member C), and *MGAT4A* (α-1,3-mannosyl-glycoprotein 4-ß-*N*-acetylglucosaminyltransferase A) were each targeted by 3 different miRNAs (Table 4). The hsa-miR-16-5p was found to be the only common miRNA targeting these 4 genes. The complete list of target genes, their associated miRNAs and prediction scores is provided in Supplementary Table 2. TF analysis performed independently from target gene prediction revealed a significant association with *NR1H4 *(Nuclear Receptor Subfamily 1 Group H Member 4), related to hsa-miR-100-5p and hsa-miR-34a-5p (*P *< .005, FDR = 0.4931).

### Assessment of Pathway and Expression Analysis of microRNA Interactors

**Figure 4.**
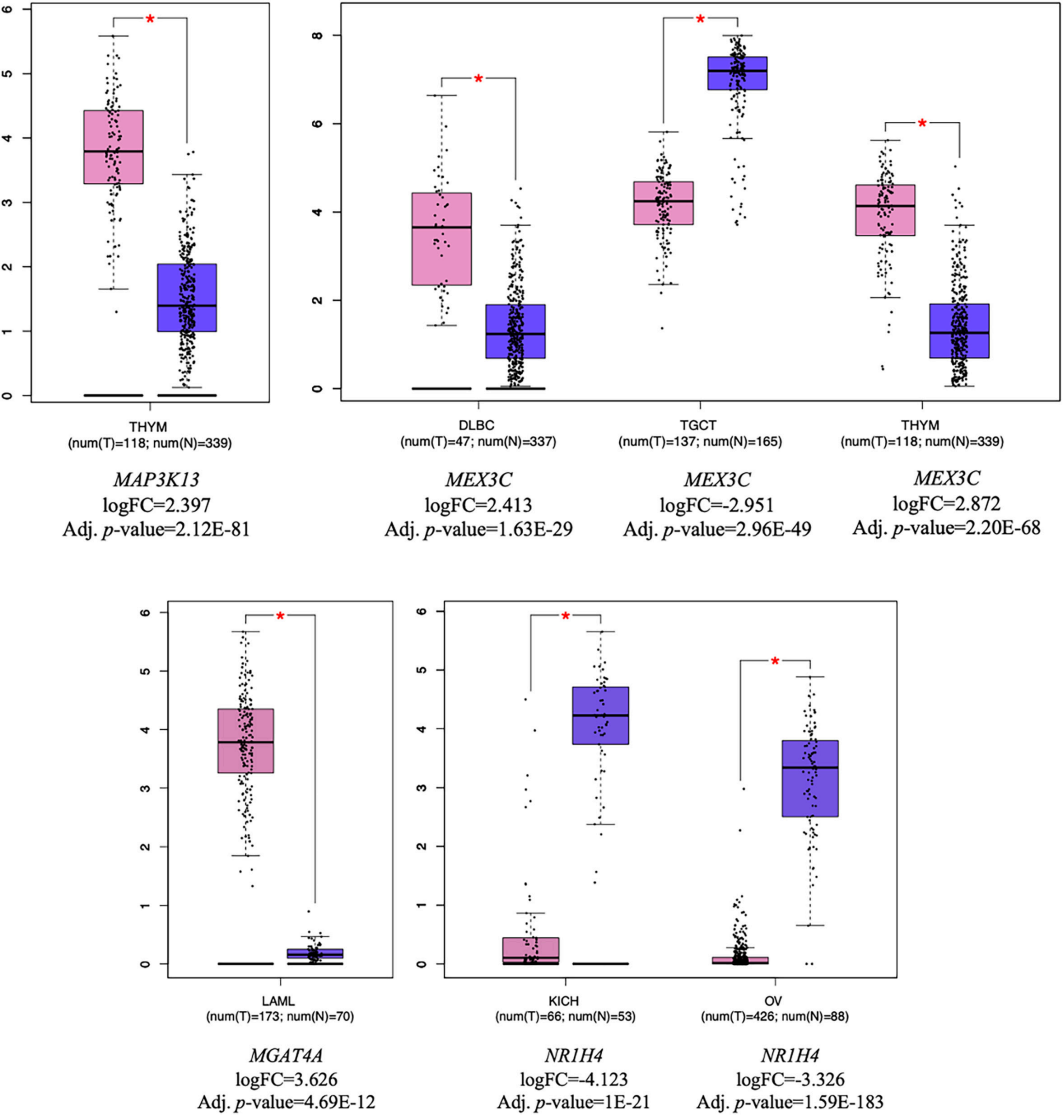
Differential expression patterns of predicted target genes and transcription factor in TCGA datasets (|log_2_FC|≥2 and *P *< .005). Boxplots illustrate the differential expression of miRNA target genes and a transcription factor in tumor versus normal tissues across TCGA cancer types. Pink boxes represent tumor samples, while purple boxes represent normal samples. Red asterisks indicate statistically significant differences. For each gene, the corresponding log_2_FC and adjusted *p*-values are provided for cancer types in which the expression was found to be significant. DLBC, diffuse large B-cell lymphoma; KICH, kidney chromophobe; LAML, acute myeloid lLeukemia; OV, ovarian serous cystadenocarcinoma; TCGA, The Cancer Genome Atlas; TGCT, testicular germ cell tumors; THYM, thymoma.

Pathway analysis revealed 6 KEGG pathways, the most significant being the “circadian rhythm” pathway (*P *< .05). *CLOCK* and *MGAT4A* were found to interact with 2 different KEGG pathways. Detailed information on the identified pathways is provided in Table 5. Expression analysis of target genes and TFs across various cancer types was performed using GEPIA2. Thymoma (THYM) were identified as the most significantly associated with target genes and TF (n = 2) (Figure 4).

## Discussion

This study utilized an integrative bioinformatic approach to explore the miRNA interactome networks in HPV-16 infection, shedding light on the molecular mechanisms underlying HPV-16-driven carcinogenesis. By leveraging viRBase, TCGA, and GTEx datasets, the authors identified key miRNAs, target genes, and TF contributing to HPV-16-associated tumorigenesis. Receiver operating characteristic curveand Kaplan-Meier survival analyses were utilized to assess the diagnostic and prognostic potential of the identified miRNAs and their target genes in TCGA datasets.

Notably, 8 miRNAs (hsa-miR-16-5p, hsa-miR-24-3p, hsa-miR-34a-5p, hsa-miR-100-5p, hsa-miR-125b-5p, hsa-miR-203a-3p, hsa-miR-205-5p, and hsa-miR-331-3p) were examined in TCGA and GTEx datasets and were determined to be target genes and related to TF (Table 1). The KEGG pathways and GO functional analysis of all miRNAs revealed cancer-related pathways and viral processes, respectively (*P *< .05) (Figure 1).

The authors note that hsa-miR-100-5p, hsa-miR-125b-5p, and hsa-miR-331-3p were the most significantly dysregulated across multiple cancer types (n = 11), suggesting their potential role as oncogenic regulators in HPV-related malignancies (Figure 3). Remarkably, hsa-miR-125b-5p emerged as the most significant miRNA in HPV-related cancers based on ROC analysis (Table 2) and Kaplan-Meier survival analysis (Table 3), further highlighting its clinical relevance. Wang et al^12^ found that hsa-miR-100-5p was downregulated in HPV-16 infected HFK raft cultures via Northern blot analysis. The decreased expression of hsa-miR-100 may be linked to the activity of viral oncoproteins E6 and E7, which target multiple cellular transcription factors12. Additionally, HPV L2 protein directly inactivates hsa-miR-125b, promoting koilocyte formation, while exogenous application of hsa-miR-125b significantly suppresses HPV DNA synthesis.^27^ Significantly, hsa-miR-125b is not decreased in cervical cancer, possibly due to the limited expression of L2 in these tumors.^27^ Furthermore, *p53*, a key component of HPV infection, is suppressed by hsa-miR-125b.^27,29^ The other miRNA, hsa-miR-331-3p, suppressed cervical cancer cell proliferation by targeting *NRP2 *(Neuropilin 2), leading to the downregulation of HPV16 E6/E7 oncoproteins and promoting keratinocyte differentiation. This suggests a potential therapeutic role for miR-331-3p in HPV-related cervical cancer.^13^ These findings emphasize the critical roles of hsa-miR-100-5p, hsa-miR-125b-5p, and hsa-miR-331-3p in HPV-related carcinogenesis and their potential therapeutic biomarkers.

The authors’ analyses revealed that hsa-miR-16-5p, hsa-miR-24-3p, hsa-miR-34a-5p, hsa-miR-203a-3p, and hsa-miR-205-5p also play pivotal roles in HPV infection-related molecular mechanisms. HPV-16 E6/E7 oncoproteins disrupt the expression of hsa-miR-24-3p, hsa-miR-203-3p and hsa-miR-205-5p in HFKs. Among these, hsa-miR-205-5p expression is specifically regulated by E7, while hsa-miR-24-3p directly targets the cell cycle regulator *p27*. The expression of hsa-miR-203a-3p is reduced when p53 is compromised either by E6 or through *p53* knockout, thereby affecting both cellular differentiation and DNA damage response.^11,26^ Human papillomavirus E6 and E7 also upregulated *ESM1* (Endothelial Cell Specific Molecule 1) in CESC by inhibiting hsa-miR-205-5p, with *ESM1* promoting glycolysis through the Akt/mTOR pathway. Its suppression reduced *HIF-1α* (Hypoxia Inducible Factor 1 Subunit Alpha) and glycolytic enzyme expression, contributing to carcinogenesis.^30^ In the authors’ analysis, hsa-miR-16-5p, which targets 4 genes (*CLOCK, MAP3K13, MEX3C*, and *MGAT4A*), has been implicated in HPV-related carcinogenesis. Jarych et al^31^ demonstrated that elevated hsa-miR-16-5p served as an independent prognostic factor for poor overall survival in high-risk HPV-16 and/or HPV-18 infection in epithelial ovarian neoplasms.^31^ The elevated expression of hsa-miR-16 was linked to the activity of viral oncoproteins E6 and/or E7, which are known to target numerous cellular TFs. Additionally, the increased expression of hsa-miR-16 in cervical intraepithelial neoplasia and cervical cancer tissues infected with high-risk HPV was found to be consistent with HPV-16 infection.^12^ Furthermore, Wang et al demonstrated reduced hsa-miR-34a expression in HPV-related cervical cancer, attributed to E6-mediated *p53* destabilization.^28^ This downregulation is attributed to the viral oncoprotein E6, which destabilizes the tumor suppressor *p53*, a known transactivator of hsa-miR-34a. Li et al^32^ highlighted that HPV-16 E6 downregulates pri-miR-34a expression via p53-dependent pathway, indicating its early role in HPV-related carcinogenesis. Similarly, Gao et al^33^ showed reduced miR-34a-5p and upregulated *JAG1* (Jagged Canonical Notch Ligand 1) in HPV-16-infected keratinocytes, with miR-34a-5p targeting *JAG1* to regulate the Notch1 pathway. These findings highlight the critical role of HPV-16 oncoproteins in modulating miRNA expression and their contribution to carcinogenesis.

The authors observed that BRCA and LUSC showed the highest number of significant miRNA alterations (n = 7) (Figure 3, Supplementary Figure 1). Two different meta-analyses and large-scale systematic reviews showed that HPV infection, particularly with HPV-16 and HPV-18 infection, significantly elevated the risk of lung and breast cancer. Moreover, CESC, COAD, and READ showed the highest number of altered miRNA interactions (AUC ≥ 0.90) (Table 2), while UVM exhibited the highest number of prognostic miRNAs in Kaplan-Meier analysis (*P*< .005) (Table 3). Bodaghi et al^34^ demonstrated that HPV-16 was the most prevalent type in colorectal tissues. Cervical and endocervical cancers is the most prevalent HPV-related disease, and approximately 99.7% of cervical cancer cases are caused by persistent genital high-risk HPV infection.^35^ Several reports suggest a possible association between HPV infection and colorectal cancer (CRC) development. The population-based cohort study demonstrated a significantly higher risk of CRC among individuals with HPV infection, indicating a potential link.^36^ Similarly, Javadi et al^37^ reported frequent detection of HPV-16 DNA and its E6/E7 oncoproteins in CRC tissues, urine, and serum-derived exosomes, supporting both its oncogenic role and the feasibility of non-invasive diagnostics. Only one study identified HPV-related infection in UVM cell lines, suggesting that HPV might be involved in ocular melanoma pathogenesis.^38^ Although UVM was the only type to show significance in Kaplan-Meier analysis, to the authors’ knowledge, there is no relevant information in the literature regarding HPV-16 in relation to UVM. This highlights that HPV-16 infection has a varying influence across different cancer types, and its potential impact on diagnostics, prognosis, and theranostics may offer valuable insights for future research.

The authors found that* MAP3K13* was targeted by 4 miRNAs (hsa-miR-16-5p, hsa-miR-125b-5p, hsa-miR-203a-3p, hsa-miR-205-5p), while *MGAT4A*, *MEX3C*, and *CLOCK* were each targeted by 3 miRNAs (Table 4). Hsa-miR-16-5p was the only common miRNA targeting all 4 genes. Smokers with HPV-positive oropharyngeal squamous cell carcinoma exhibited poorer overall survival compared to non-smokers, particularly in cases harboring *MAP3K13* amplification.^39^ Additionally, Ramirez-Salazar et al^40^ found that *MGAT4A *was highly elevated in C-33A cells following HPV-16 E2 expression, indicating a potential role in HPV-mediated cellular changes. On the other hand, *MEX3C*, an RNA-binding protein, was upregulated in hepatocellular carcinoma compared to normal tissues, and its high expression correlated with poor prognosis.^41^ *MEX3C *also promoted bladder carcinogenesis by modulating lipid metabolism via the c-Jun NH2-terminal kinase (JNK) pathway and could contribute to lung adenocarcinoma tumorigenesis through the ubiquitylation of RUNX Family Transcription Factor 3 (*RUNX3*).^42^ *CLOCK*, one of the predicted target genes of HPV-associated miRNAs, may represent a novel player in HPV-associated carcinogenesis. The authors’ TF analysis revealed a significant association with *NR1H4*, a member of the superfamily of nuclear receptors linked to hsa-miR-100-5p and hsa-miR-34a-5p (*P *< .005). The *NR1H4* has recently been identified as a molecular mediator regulating tumorigenesis,^43^ though its role in HPV-mediated oncogenesis remains unexplored. The authors hypothesize that *CLOCK* and *NR1H4* may individually contribute to HPV-16-driven carcinogenesis, which requires experimental validation; to the authors’ knowledge, no prior studies have investigated their interplay in viral carcinogenesis.

In summary, the authors have provided further evidence of diverse molecular mechanisms underlying HPV-associated cancers, revealing critical insights into the regulatory networks disrupted by HPV-16. The identification of key miRNAs, downstream targets, and TF, such as hsa-miR-100-5p, hsa-miR-125b-5p, *MAP3K13,* and *NR1H4*, highlights their potential roles in HPV-mediated carcinogenesis. This interactome profile not only enhances the authors’ understanding of the HPV-16 infection but also offers promising strategies for pioneering biomarkers and therapeutic targets.

This study has several limitations due to the bioinformatic design, but it provides valuable insight into the role of miRNA-mediated interaction networks in HPV-16-induced carcinogenesis. Firstly, the authors’ results are promising and warrant validation by wet lab experiments to elucidate miRNA interactome networks in HPV-16-derived carcinogenesis. Secondly, the study primarily focuses on HPV-16, and the findings may not fully apply to other high-risk HPV subtypes, such as HPV-18, HPV-31, and HPV-45. Further research is needed to explore miRNA interactome networks across different HPV types.

In conclusion, integrative bioinformatic analysis revealed novel miRNA-based interactions implicated in HPV-16-related carcinogenesis. Following validation of the expression levels and clinical significance of these miRNAs, candidate target genes, and TF in multiple databases, the authors propose that they could serve as a potential biomarker panel for the prognostic evaluation and therapeutic targeting of HPV-16-associated tumorigenesis. However, further research is essential to fully elucidate these molecular interactions and validate their clinical applicability, paving the way for innovative translational strategies to enhance HPV-associated cancer management.

### Author Contirbutions:

Concept – B.D.; Design – B.D.; Supervision – B.D.; Resources – B.D.; Materials – B.D.; Data Collection and/or Processing – B.D.; Analysis and/or Interpretation – B.D.; Literature Search – B.D.; Writing Manuscript – B.D.; Critical Review – B.D.

## Supplementary Materials

Supplementary Material

## Figures and Tables

**Table 1.** microRNA Interactome Networks Based on Host-Virus Interaction in Human Papillomavirus-16 Infection

HPV-16 mRNA/Protein	Viral Interactor Expression	Host miRNA	Host Interactor Expression	Target Region	Cell Types	Interaction Score	Reference
E7	ND	hsa-miR-205-5p	Down	ND	HFK cells	0.993	McKenna et al., 2014
E7	Down	hsa-miR-203a-3p	Up	3’UTR	H1299 and HFK cells	0.993	McKenna et al., 2010
E6	Down	hsa-miR-203a-3p	Up	3’UTR	H1299 and HFK cells	0.982	McKenna et al., 2010
E7	ND	hsa-miR-24-3p	Up	ND	HFK cells	0.982	McKenna et al., 2014
E6	ND	hsa-miR-24-3p	Up	ND	HFK cells	0.982	McKenna et al., 2014
L2 capsid	ND	hsa-miR-125b-5p	ND	3’UTR	HEK cells	0.953	Nuovo et al., 2010
E7	ND	hsa-miR-100-5p	Down	ND	HFK and HVK cells	0.949	Wang et al., 2014
E7	ND	hsa-miR-16-5p	Up	ND	HFK and HVK cells	0.949	Wang et al., 2014
E6	ND	hsa-miR-16-5p	Up	ND	HFK and HVK cells	0.949	Wang et al., 2014
E6	ND	hsa-miR-34a-5p	Down	5’UTR	CaSki, HeLa, HCT116 cells	0.881	Wang et al., 2009
E7	Down	hsa-miR-331-3p	ND	ND	SKG-II, HCS-2, HeLa cells	0.731	Fujii et al., 2016

HFK, human foreskin keratinocytes; HVK, human vaginal keratinocytes; ND, not determined; UTR, untranslated region.

**Table 2.** ROC Analysis of microRNA Diagnostic Potential and Interaction Networks Across Cancer Types (AUC ≥ 0.90)

	hsa-miR-16-5p	hsa-miR-24-3p	hsa-miR-34a-5p	hsa-miR-100-5p	**hsa-miR-125b-5p**	hsa-miR-203a-3p	hsa-miR-205-5p	hsa-miR-331-3p
BLCA (T = 409, N = 19)				0.95	0.95			
BRCA (T = 1078, N =1 04)				0.93	0.95			
**CESC **(T = 307, N = 3)	0.99			0.99	0.98	0.97	0.95	0.93
CHOL (T = 36, N = 9)		0.90			0.94			
**COAD **(T = 444, N = 8)	1	1	0.99		0.98	1		0.94
HNSC (T = 523, N = 44)				0.96	0.90			
KICH (T = 66, N = 25)	0.90							
KIRC (T = 516, N = 71)	0.95				0.94			
KIRP (T = 291, N = 34)			0.91					
LUSC (T = 478, N = 45)			0.91	0.98	0.90		0.97	
PCPG (T = 179, N = 3)		0.98	0.96	0.98				
**READ **(T = 161, N = 3)	1	1	1		1	1		0.96
SKCM (T = 97, N = 2)					0.91			
THCA (T = 506, N = 59)			0.97					
UCEC (T = 538, N = 33)				0.99	0.98		0.93	

The highlighted miRNAs or cancer types in bold represent the most significant. BLCA, bladder urothelial carcinoma; BRCA, breast invasive carcinoma; CESC; cervical and endocervical cancers; CHOL, cholangiocarcinoma; COAD, colon adenocarcinoma; HNSC, head and neck squamous cell carcinoma; KICH, kidney chromophobe; KIRC, kidney renal clear cell carcinoma; KIRP, kidney renal papillary cell carcinoma; LUSC, lung squamous cell carcinoma; N, normal; PCPG, pheochromocytoma and paraganglioma; READ, rectum adenocarcinoma; SKCM, skin cutaneous melanoma; T, tumor; THCA, thyroid carcinoma; UCEC, uterine corpus endometrial carcinoma.

**Table 3.** Kaplan-Meier Survival Analysis of miRNAs and Their Prognostic Relevance in Cancer (*P*< .005)

		hsa-miR-16-5p	hsa-miR-24-3p	hsa-miR-34a-5p	hsa-miR-100-5p	**hsa-miR-125b-5p**	hsa-miR-203a-3p
ACC (T = 80)	HR			0.28 (0.14-0.59)		0.24 (0.12-0.5)	
	*P*			1.10E-03		3.57E-04	
BLCA (T = 409)	HR					1.73 (1.29-2.32)	
	*P*					3.03E-04	
KIRP (T = 291)	HR			0.27 (0.15-0.49)			
	*P*			9.96E-05			
LAML (T = 188)	HR	1.77 (1.21-2.57)			0.58 (0.4-0.84)		
	*P*	2.01E-03			3.38E-03		
LGG (T = 512)	HR	1.91 (1.34-2.72)	1.94 (1.36-2.76)				
	*P*	4.23E-04	2.68E-04				
LIHC (T = 372)	HR				0.56 (0.4-0.8)	0.56 (0.39-0.79)	
	*P*				1.2E-03	9.08E-04	
PAAD (T = 178)	HR		1.83 (1.22-2.76)				1.96 (1.3-2.95)
	*p*-value		3.48E-03				1.09E-03
**UVM **(T = 80)	HR		3.69 (1.62-8.42)			0.22 (0.1-0.52)	0.26 (0.11-0.59)
	*P*		3E-03			3.40E-04	1.31E-03

The highlighted miRNAs or cancer types in bold represent the most significant. ACC, adrenocortical carcinoma; BLCA, bladder urothelial carcinoma; HR, hazard ratio; KIRP, kidney renal papillary cell carcinoma; LAML, acute myeloid leukemia; LGG, lower grade glioma; LIHC, liver hepatocellular carcinoma; PAAD, pancreatic adenocarcinoma; T, tumor; UVM, uveal melanoma.

**Table 4.** Predicted Target Genes Regulated by at least 3 microRNA Interactors (score ≥ 90)

Gene Symbol	# miRNA	miRNA	Score
*MAP3K13*	4	hsa-miR-16-5p	90
		hsa-miR-125b-5p	90
		hsa-miR-203a-3p	96
		hsa-miR-205-5p	97
*CLOCK*	3	hsa-miR-16-5p	91
		hsa-miR-34a-5p	90
		hsa-miR-203a-3p	97
*MEX3C*	3	hsa-miR-16-5p	92
		hsa-miR-34a-5p	95
		hsa-miR-203a-3p	99
*MGAT4A*	3	hsa-miR-16-5p	99
		hsa-miR-24-3p	94
		hsa-miR-34a-5p	99

CLOCK, clock circadian regulator; MAP3K13, mitogen-activated protein kinase Kinase 1; MEX3C, Mex-3 RNA-binding family member C; MGAT4A, alpha-1,3-mannosyl-glycoprotein 4-beta-*N*-acetylglucos-aminyltransferase A.

**Table 5.** Pathway Enrichment Analysis of Target Genes and Transcription Factor (*P *< .05)

KEGG Pathway	*P*	Adjusted *P*	Related Gene/TF
Circadian rhythm	.008	.025	*CLOCK*
Various types of N-glycan biosynthesis	.010	.025	*MGAT4A*
N-Glycan biosynthesis	.012	.025	*MGAT4A*
Bile secretion	.022	.034	*NR1H4*
Dopaminergic synapse	.033	.039	*CLOCK*

CLOCK, clock circadian regulator; KEGG, Kyoto Encyclopedia of Genes and Genomes; MGAT4A, alpha-1,3-mannosyl-glycoprotein 4-beta-*N*-acetylglucos-aminyltransferase A; NR1H4, 2 Nuclear Receptor Subfamily 1 Group H Member 4.

## Data Availability

The data that support the findings of this study are available on request from the corresponding author.
