# Supplementary material for: Integrative Bioinformatic Approach for microRNA Interactome Networks in Human Papillomavirus-16 Infection
Source: Eurasian J Med. 2025 Sep 30;57(3):e25817. doi: 10.5152/eurasianjmed.2025.25817 (PMC12621638; doi:10.5152/eurasianjmed.2025.25817)
Supplement: Supplementary Material [file supplementary_material.pdf]

Supplementary Table 1. Comprehensive miRNA-pathway interaction in HPV-16 infection

Term	Genes	# Target Genes	# miRNAs	miRNAs	Merged *p*-value	Merged FDR
Proteoglycans in cancer	220	56.40.10.36.15.28.15	7	hsa-miR-16-5p, hsa-miR-24-3p, hsa-miR-34a-5p, hsa-miR-100-5p, hsa-miR-125b-5p, hsa-miR-203a-3p, hsa-miR-331-3p	1.396E-28	2.109E-26
Pathways in cancer	555	124.82.61.30.48	5	hsa-miR-16-5p, hsa-miR-24-3p, hsa-miR-34a-5p, hsa-miR-203a-3p, hsa-miR-125b-5p	1.592E-25	1.202E-23
Cell cycle	129	43.30.25.11.17	5	hsa-miR-16-5p, hsa-miR-24-3p, hsa-miR-34a-5p, hsa-miR-203a-3p, hsa-miR-125b-5p	2.031E-24	1.022E-22
Focal adhesion	213	51.35.36.14.24.18	6	hsa-miR-16-5p, hsa-miR-24-3p, hsa-miR-34a-5p, hsa-miR-203a-3p, hsa-miR-125b-5p, hsa-miR-331-3p	6.029E-23	2.276E-21
Prostate cancer	101	33.19.22.5.13.8	6	hsa-miR-16-5p, hsa-miR-24-3p, hsa-miR-34a-5p, hsa-miR-205-5p, hsa-miR-125b-5p, hsa-miR-331-3p	2.209E-19	6.670E-18
Hippo signaling pathway	164	39.29.22.16.26	5	hsa-miR-16-5p, hsa-miR-24-3p, hsa-miR-34a-5p, hsa-miR-203a-3p, hsa-miR-125b-5p	4.727E-19	1.190E-17
PI3K-Akt signaling pathway	372	69.54.46.22.26	5	hsa-miR-16-5p, hsa-miR-24-3p, hsa-miR-34a-5p, hsa-miR-203a-3p, hsa-miR-331-3p	1.115E-18	2.406E-17
Bacterial invasion of epithelial cells	80	26.19.17.10.12	5	hsa-miR-16-5p, hsa-miR-24-3p, hsa-miR-34a-5p, hsa-miR-203a-3p, hsa-miR-125b-5p	1.895E-18	3.577E-17
Colorectal cancer	88	25.20.16.13.13	5	hsa-miR-16-5p, hsa-miR-24-3p, hsa-miR-34a-5p, hsa-miR-125b-5p, hsa-miR-331-3p	3.841E-18	6.444E-17
Regulation of actin cytoskeleton	224	48.36.35.32	4	hsa-miR-16-5p, hsa-miR-24-3p, hsa-miR-34a-5p, hsa-miR-125b-5p	9.625E-18	1.453E-16
Shigellosis	268	70.37.31.16.15	5	hsa-miR-16-5p, hsa-miR-24-3p, hsa-miR-34a-5p, hsa-miR-203a-3p, hsa-miR-331-3p	4.482E-16	6.153E-15
Adherens junction	79	29.15.21	3	hsa-miR-16-5p, hsa-miR-24-3p, hsa-miR-34a-5p	5.002E-16	6.294E-15
Pancreatic cancer	78	24.19.16.10	4	hsa-miR-16-5p, hsa-miR-24-3p, hsa-miR-34a-5p, hsa-miR-331-3p	8.496E-16	9.869E-15
MAPK signaling pathway	329	68.42.42.35.19	5	hsa-miR-16-5p, hsa-miR-24-3p, hsa-miR-34a-5p, hsa-miR-125b-5p, hsa-miR-331-3p	1.815E-15	1.828E-14
Viral carcinogenesis	265	68.35.36.27	4	hsa-miR-16-5p, hsa-miR-24-3p, hsa-miR-34a-5p, hsa-miR-125b-5p	1.698E-15	1.828E-14
Hepatocellular carcinoma	177	35.34.28.20.12	5	hsa-miR-16-5p, hsa-miR-24-3p, hsa-miR-34a-5p, hsa-miR-125b-5p, hsa-miR-331-3p	3.900E-15	3.680E-14
Hepatitis B	177	49.31.28	3	hsa-miR-16-5p, hsa-miR-24-3p, hsa-miR-34a-5p	4.848E-15	4.306E-14
Cellular senescence	219	46.37.35.13	4	hsa-miR-16-5p, hsa-miR-24-3p, hsa-miR-34a-5p, hsa-miR-331-3p	1.503E-14	1.261E-13
Signaling pathways regulating pluripotency of stem cells	156	42.24.17.17	4	hsa-miR-16-5p, hsa-miR-24-3p, hsa-miR-203a-3p, hsa-miR-125b-5p	2.168E-14	1.723E-13
Salmonella infection	277	64.39.30.27.17	5	hsa-miR-16-5p, hsa-miR-24-3p, hsa-miR-34a-5p, hsa-miR-125b-5p, hsa-miR-331-3p	9.160E-14	6.916E-13
TGF-beta signaling pathway	103	33.16.13.12.8	5	hsa-miR-16-5p, hsa-miR-24-3p, hsa-miR-34a-5p, hsa-miR-203a-3p, hsa-miR-331-3p	9.646E-14	6.936E-13
EGFR tyrosine kinase inhibitor resistance	82	19.16.17.5.9	5	hsa-miR-16-5p, hsa-miR-24-3p, hsa-miR-34a-5p, hsa-miR-205-5p, hsa-miR-331-3p	1.087E-13	7.460E-13

(*Continued*)

**Supplementary Table 1.** Comprehensive miRNA-pathway interaction in HPV-16 infection (*Continued*)						
Term	Genes	# Target Genes	# miRNAs	miRNAs	Merged *p*-value	Merged FDR
Yersinia infection	147	36.22.24.12.17	5	hsa-miR-16-5p, hsa-miR-24-3p, hsa-miR-34a-5p, hsa-miR-203a-3p, hsa-miR-125b-5p	1.568E-13	1.029E-12
Chronic myeloid leukemia	79	22.18.20	3	hsa-miR-16-5p, hsa-miR-24-3p, hsa-miR-34a-5p	2.149E-13	1.352E-12
Endocrine resistance	118	31.22.20.11	4	hsa-miR-16-5p, hsa-miR-24-3p, hsa-miR-34a-5p, hsa-miR-331-3p	3.173E-13	1.916E-12
Thyroid hormone signaling pathway	137	31.25.24.11	4	hsa-miR-16-5p, hsa-miR-24-3p, hsa-miR-34a-5p, hsa-miR-331-3p	7.674E-13	4.457E-12
Renal cell carcinoma	70	24.13.18	3	hsa-miR-16-5p, hsa-miR-24-3p, hsa-miR-34a-5p	9.638E-13	5.390E-12
Neurotrophin signaling pathway	124	36.21.20.15	4	hsa-miR-16-5p, hsa-miR-24-3p, hsa-miR-34a-5p, hsa-miR-125b-5p	1.329E-12	7.169E-12
Bladder cancer	43	14.11.12.4	4	hsa-miR-16-5p, hsa-miR-24-3p, hsa-miR-34a-5p, hsa-miR-205-5p	2.007E-12	1.045E-11
Central carbon metabolism in cancer	74	17.13.16.11.9	5	hsa-miR-16-5p, hsa-miR-24-3p, hsa-miR-34a-5p, hsa-miR-125b-5p, hsa-miR-331-3p	5.732E-12	2.885E-11
Rap1 signaling pathway	214	54.27.32	3	hsa-miR-16-5p, hsa-miR-24-3p, hsa-miR-34a-5p	9.635E-12	4.693E-11
Human papillomavirus infection	406	85.53.43	3	hsa-miR-16-5p, hsa-miR-24-3p, hsa-miR-34a-5p	5.974E-11	2.819E-10
Endometrial cancer	61	16.12.11.11.7	5	hsa-miR-16-5p, hsa-miR-24-3p, hsa-miR-34a-5p, hsa-miR-125b-5p, hsa-miR-331-3p	1.424E-10	6.516E-10
FoxO signaling pathway	139	37.21.19.10	4	hsa-miR-16-5p, hsa-miR-24-3p, hsa-miR-34a-5p, hsa-miR-203a-3p	2.633E-10	1.169E-09
MicroRNAs in cancer	334	38.45.9	3	hsa-miR-24-3p, hsa-miR-34a-5p, hsa-miR-205-5p	3.683E-10	1.589E-09
Autophagy - animal	146	45.11	2	hsa-miR-16-5p, hsa-miR-331-3p	4.087E-10	1.714E-09
p53 signaling pathway	75	26.14.13	3	hsa-miR-16-5p, hsa-miR-24-3p, hsa-miR-34a-5p	5.223E-10	2.132E-09
Breast cancer	163	37.25.23.11	4	hsa-miR-16-5p, hsa-miR-24-3p, hsa-miR-34a-5p, hsa-miR-331-3p	5.535E-10	2.200E-09
Non-small cell lung cancer	81	19.15.15.11.7	5	hsa-miR-16-5p, hsa-miR-24-3p, hsa-miR-34a-5p, hsa-miR-125b-5p, hsa-miR-331-3p	6.325E-10	2.449E-09
Protein processing in endoplasmic reticulum	194	52.21.13	3	hsa-miR-16-5p, hsa-miR-34a-5p, hsa-miR-203a-3p	6.997E-10	2.642E-09
HIF-1 signaling pathway	112	20.18.14.9	4	hsa-miR-24-3p, hsa-miR-34a-5p, hsa-miR-125b-5p, hsa-miR-331-3p	9.154E-10	3.371E-09
Axon guidance	186	38.31.20.21	4	hsa-miR-16-5p, hsa-miR-24-3p, hsa-miR-34a-5p, hsa-miR-125b-5p	3.552E-09	1.277E-08
Oocyte meiosis	134	38.20	2	hsa-miR-16-5p, hsa-miR-34a-5p	1.017E-08	3.570E-08
Hepatitis C	173	40.27.22	3	hsa-miR-16-5p, hsa-miR-24-3p, hsa-miR-34a-5p	1.599E-08	5.486E-08
Ubiquitin mediated proteolysis	142	37.16.12	3	hsa-miR-16-5p, hsa-miR-34a-5p, hsa-miR-203a-3p	1.860E-08	6.241E-08
AMPK signaling pathway	130	28.20.15.15.10	5	hsa-miR-16-5p, hsa-miR-24-3p, hsa-miR-34a-5p, hsa-miR-125b-5p, hsa-miR-331-3p	2.230E-08	7.320E-08
Insulin signaling pathway	153	31.23.22.10	4	hsa-miR-16-5p, hsa-miR-24-3p, hsa-miR-34a-5p, hsa-miR-331-3p	2.615E-08	8.403E-08
Fluid shear stress and atherosclerosis	149	38.26	2	hsa-miR-16-5p, hsa-miR-24-3p	3.380E-08	1.063E-07
AGE-RAGE signaling pathway in diabetic complications	115	27.21.17	3	hsa-miR-16-5p, hsa-miR-24-3p, hsa-miR-34a-5p	4.248E-08	1.309E-07

(*Continued*)

Supplementary Table 1. Comprehensive miRNA-pathway interaction in HPV-16 infection (*Continued*)

Term	Genes	# Target Genes	# miRNAs	miRNAs	Merged *p*-value	Merged FDR
Kaposi sarcoma-associated herpesvirus infection	245	48.33.31	3	hsa-miR-16-5p, hsa-miR-24-3p, hsa-miR-34a-5p	6.856E-08	2.071E-07
Apoptosis	151	34.22.22	3	hsa-miR-16-5p, hsa-miR-24-3p, hsa-miR-34a-5p	8.656E-08	2.563E-07
Longevity regulating pathway	105	24.17.11	3	hsa-miR-16-5p, hsa-miR-24-3p, hsa-miR-331-3p	1.278E-07	3.712E-07
Melanoma	76	21.16	2	hsa-miR-16-5p, hsa-miR-34a-5p	1.477E-07	4.208E-07
RNA transport	199	42.24.12	3	hsa-miR-16-5p, hsa-miR-125b-5p, hsa-miR-331-3p	1.601E-07	4.476E-07
ErbB signaling pathway	86	15.14.13	3	hsa-miR-24-3p, hsa-miR-34a-5p, hsa-miR-125b-5p	2.124E-07	5.831E-07
Small cell lung cancer	100	24.19.14	3	hsa-miR-16-5p, hsa-miR-24-3p, hsa-miR-34a-5p	2.682E-07	7.231E-07
Gastric cancer	162	33.25.22	3	hsa-miR-16-5p, hsa-miR-24-3p, hsa-miR-34a-5p	2.975E-07	7.880E-07
Transcriptional misregulation in cancer	206	27.25.14	3	hsa-miR-24-3p, hsa-miR-34a-5p, hsa-miR-203a-3p	3.897E-07	1.014E-06
Wnt signaling pathway	173	39.25	2	hsa-miR-16-5p, hsa-miR-34a-5p	4.761E-07	1.219E-06
Growth hormone synthesis. secretion and action	129	33.19	2	hsa-miR-16-5p, hsa-miR-34a-5p	6.265E-07	1.577E-06
Alcoholism	195	42.27	2	hsa-miR-16-5p, hsa-miR-34a-5p	8.451E-07	2.058E-06
Human cytomegalovirus infection	306	55.37.36	3	hsa-miR-16-5p, hsa-miR-24-3p, hsa-miR-34a-5p	8.415E-07	2.058E-06
Long-term depression	64	19.13	2	hsa-miR-16-5p, hsa-miR-34a-5p	8.595E-07	2.060E-06
Endocytosis	311	62.38.16	3	hsa-miR-16-5p, hsa-miR-24-3p, hsa-miR-331-3p	8.873E-07	2.063E-06
Parathyroid hormone synthesis. secretion and action	118	26.21	2	hsa-miR-16-5p, hsa-miR-34a-5p	8.881E-07	2.063E-06
Relaxin signaling pathway	138	19.20.10	3	hsa-miR-24-3p, hsa-miR-34a-5p, hsa-miR-331-3p	1.109E-06	2.538E-06
VEGF signaling pathway	61	15.13.11	3	hsa-miR-16-5p, hsa-miR-24-3p, hsa-miR-34a-5p	1.355E-06	3.054E-06
TNF signaling pathway	131	33.18	2	hsa-miR-16-5p, hsa-miR-34a-5p	2.585E-06	5.658E-06
Progesterone-mediated oocyte maturation	104	26.17	2	hsa-miR-16-5p, hsa-miR-34a-5p	2.553E-06	5.658E-06
Measles	161	34.24	2	hsa-miR-16-5p, hsa-miR-34a-5p	2.628E-06	5.668E-06
Sphingolipid signaling pathway	133	33.18	2	hsa-miR-16-5p, hsa-miR-34a-5p	4.080E-06	8.678E-06
Acute myeloid leukemia	69	19.13	2	hsa-miR-16-5p, hsa-miR-34a-5p	4.752E-06	9.966E-06
Long-term potentiation	71	16	1	hsa-miR-34a-5p	5.084E-06	1.052E-05
Fc gamma R-mediated phagocytosis	101	23.16.14	3	hsa-miR-16-5p, hsa-miR-24-3p, hsa-miR-34a-5p	1.110E-05	2.266E-05
Tight junction	182	41.25	2	hsa-miR-16-5p, hsa-miR-24-3p	1.268E-05	2.552E-05
Cushing syndrome	176	36.24	2	hsa-miR-16-5p, hsa-miR-34a-5p	1.311E-05	2.605E-05
Prolactin signaling pathway	73	19.12.11	3	hsa-miR-16-5p, hsa-miR-24-3p, hsa-miR-34a-5p	1.418E-05	2.781E-05
cGMP-PKG signaling pathway	175	37.23	2	hsa-miR-16-5p, hsa-miR-34a-5p	1.472E-05	2.849E-05
Pathogenic Escherichia coli infection	222	48.22	2	hsa-miR-16-5p, hsa-miR-125b-5p	1.598E-05	3.016E-05
Glioma	79	18.15	2	hsa-miR-16-5p, hsa-miR-34a-5p	1.585E-05	3.016E-05
PD-L1 expression and PD-1 checkpoint pathway in cancer	101	18.14	2	hsa-miR-24-3p, hsa-miR-34a-5p	2.777E-05	5.178E-05
Lysine degradation	69	20.12	2	hsa-miR-16-5p, hsa-miR-24-3p	2.986E-05	5.499E-05
mRNA surveillance pathway	108	14.9	2	hsa-miR-125b-5p, hsa-miR-331-3p	3.442E-05	6.262E-05

(*Continued*)

Supplementary Table 1. Comprehensive miRNA-pathway interaction in HPV-16 infection (*Continued*)

Term	Genes	# Target Genes	# miRNAs	miRNAs	Merged *p*-value	Merged FDR
Amyotrophic lateral sclerosis	408	70.12	2	hsa-miR-16-5p, hsa-miR-100-5p	4.100E-05	7.370E-05
Choline metabolism in cancer	106	16.8	2	hsa-miR-34a-5p, hsa-miR-331-3p	4.854E-05	8.623E-05
Ras signaling pathway	241	45.29	2	hsa-miR-16-5p, hsa-miR-34a-5p	5.091E-05	8.874E-05
Oxytocin signaling pathway	161	24	1	hsa-miR-34a-5p	5.113E-05	8.874E-05
Apelin signaling pathway	140	30.19	2	hsa-miR-16-5p, hsa-miR-34a-5p	5.601E-05	9.611E-05
Notch signaling pathway	63	19.9	2	hsa-miR-16-5p, hsa-miR-34a-5p	6.254E-05	1.038E-04
Melanogenesis	107	24.16	2	hsa-miR-16-5p, hsa-miR-34a-5p	6.210E-05	1.038E-04
Chagas disease	116	29.14	2	hsa-miR-16-5p, hsa-miR-34a-5p	6.246E-05	1.038E-04
Insulin resistance	124	18.17	2	hsa-miR-24-3p, hsa-miR-34a-5p	1.164E-04	1.910E-04
Various types of N-glycan biosynthesis	40	13.7	2	hsa-miR-16-5p, hsa-miR-34a-5p	1.642E-04	2.665E-04
Basal cell carcinoma	66	12	1	hsa-miR-125b-5p	1.708E-04	2.743E-04
Vasopressin-regulated water reabsorption	47	14.9	2	hsa-miR-16-5p, hsa-miR-24-3p	2.061E-04	3.275E-04
Vibrio cholerae infection	60	16.9	2	hsa-miR-16-5p, hsa-miR-34a-5p	4.585E-04	7.211E-04
Human immunodeficiency virus 1 infection	277	51.28	2	hsa-miR-16-5p, hsa-miR-34a-5p	5.570E-04	8.671E-04
Estrogen signaling pathway	167	22	1	hsa-miR-34a-5p	6.225E-04	9.591E-04
N-Glycan biosynthesis	51	14.8	2	hsa-miR-16-5p, hsa-miR-34a-5p	6.548E-04	9.888E-04
Adrenergic signaling in cardiomyocytes	161	33.18	2	hsa-miR-16-5p, hsa-miR-34a-5p	6.545E-04	9.888E-04
Longevity regulating pathway - multiple species	79	8	1	hsa-miR-331-3p	7.061E-04	1.045E-03
JAK-STAT signaling pathway	168	22.19	2	hsa-miR-24-3p, hsa-miR-34a-5p	7.020E-04	1.045E-03
Dopaminergic synapse	143	29.17	2	hsa-miR-16-5p, hsa-miR-34a-5p	7.996E-04	1.172E-03
Vascular smooth muscle contraction	138	19	1	hsa-miR-34a-5p	8.359E-04	1.214E-03
Neutrophil extracellular trap formation	205	25	1	hsa-miR-34a-5p	8.731E-04	1.244E-03
C-type lectin receptor signaling pathway	116	28	1	hsa-miR-16-5p	8.702E-04	1.244E-03
Lysosome	145	33	1	hsa-miR-16-5p	9.593E-04	1.354E-03
ECM-receptor interaction	104	9	1	hsa-miR-331-3p	1.047E-03	1.464E-03
Gap junction	99	15	1	hsa-miR-34a-5p	1.077E-03	1.491E-03
GnRH signaling pathway	97	22.12	2	hsa-miR-16-5p, hsa-miR-34a-5p	1.100E-03	1.510E-03
Aldosterone synthesis and secretion	110	16	1	hsa-miR-34a-5p	1.166E-03	1.586E-03
Gastric acid secretion	80	13	1	hsa-miR-34a-5p	1.180E-03	1.591E-03
Ferroptosis	41	8	1	hsa-miR-125b-5p	1.282E-03	1.713E-03
mTOR signaling pathway	177	38	1	hsa-miR-16-5p	1.330E-03	1.762E-03
Leukocyte transendothelial migration	120	17.14	2	hsa-miR-24-3p, hsa-miR-34a-5p	1.448E-03	1.901E-03
Salivary secretion	103	15	1	hsa-miR-34a-5p	1.623E-03	2.113E-03
cAMP signaling pathway	226	26	1	hsa-miR-34a-5p	1.641E-03	2.118E-03
Cysteine and methionine metabolism	53	11	1	hsa-miR-24-3p	1.807E-03	2.312E-03
Amphetamine addiction	74	12	1	hsa-miR-34a-5p	1.837E-03	2.331E-03
Mitophagy - animal	76	12	1	hsa-miR-34a-5p	2.319E-03	2.919E-03
Platinum drug resistance	75	7	1	hsa-miR-331-3p	2.438E-03	3.042E-03
Epithelial cell signaling in *Helicobacter pylori* infection	79	20	1	hsa-miR-16-5p	2.498E-03	3.092E-03
NOD-like receptor signaling pathway	221	44	1	hsa-miR-16-5p	2.824E-03	3.466E-03
Sphingolipid metabolism	59	11	1	hsa-miR-24-3p	4.376E-03	5.247E-03
Platelet activation	136	17	1	hsa-miR-34a-5p	4.335E-03	5.247E-03
Thyroid cancer	43	8	1	hsa-miR-34a-5p	4.378E-03	5.247E-03
Glucagon signaling pathway	114	15	1	hsa-miR-34a-5p	4.423E-03	5.258E-03

(*Continued*)

Supplementary Table 1. Comprehensive miRNA-pathway interaction in HPV-16 infection (*Continued*)

Term	Genes	# Target Genes	# miRNAs	miRNAs	Merged *p*-value	Merged FDR
Pyrimidine metabolism	57	15	1	hsa-miR-16-5p	5.635E-03	6.647E-03
Apoptosis - multiple species	32	10	1	hsa-miR-16-5p	6.204E-03	7.262E-03
Necroptosis	174	23	1	hsa-miR-24-3p	6.345E-03	7.370E-03
Human T-cell leukemia virus 1 infection	352	34	1	hsa-miR-34a-5p	6.474E-03	7.463E-03
DNA replication	38	7	1	hsa-miR-34a-5p	7.968E-03	9.115E-03
Cholinergic synapse	122	15	1	hsa-miR-34a-5p	8.289E-03	9.411E-03
Other types of O-glycan biosynthesis	49	13	1	hsa-miR-16-5p	8.966E-03	1.006E-02
Huntington disease	339	60	1	hsa-miR-16-5p	8.995E-03	1.006E-02
Mannose type O-glycan biosynthesis	24	8	1	hsa-miR-16-5p	9.156E-03	1.017E-02
Inflammatory mediator regulation of TRP channels	101	13	1	hsa-miR-34a-5p	9.318E-03	1.021E-02
Alzheimer disease	426	73	1	hsa-miR-16-5p	9.330E-03	1.021E-02
Amoebiasis	113	14	1	hsa-miR-34a-5p	9.891E-03	1.074E-02
Renin secretion	72	17	1	hsa-miR-16-5p	1.061E-02	1.144E-02
Phosphatidylinositol signaling system	101	22	1	hsa-miR-16-5p	1.075E-02	1.151E-02
Insulin secretion	92	12	1	hsa-miR-34a-5p	1.105E-02	1.175E-02
RNA polymerase	40	11	1	hsa-miR-16-5p	1.187E-02	1.253E-02
Spinocerebellar ataxia	145	29	1	hsa-miR-16-5p	1.282E-02	1.344E-02
Endocrine and other factor-regulated calcium reabsorption	57	14	1	hsa-miR-16-5p	1.378E-02	1.435E-02
Fatty acid biosynthesis	24	5	1	hsa-miR-34a-5p	1.431E-02	1.474E-02
Antifolate resistance	41	11	1	hsa-miR-16-5p	1.435E-02	1.474E-02
Glycosaminoglycan biosynthesis - chondroitin sulfate / dermatan sulfate	21	7	1	hsa-miR-16-5p	1.454E-02	1.483E-02
Circadian rhythm	31	9	1	hsa-miR-16-5p	1.538E-02	1.554E-02
GnRH secretion	74	10	1	hsa-miR-34a-5p	1.543E-02	1.554E-02
Cocaine addiction	53	8	1	hsa-miR-34a-5p	1.557E-02	1.557E-02

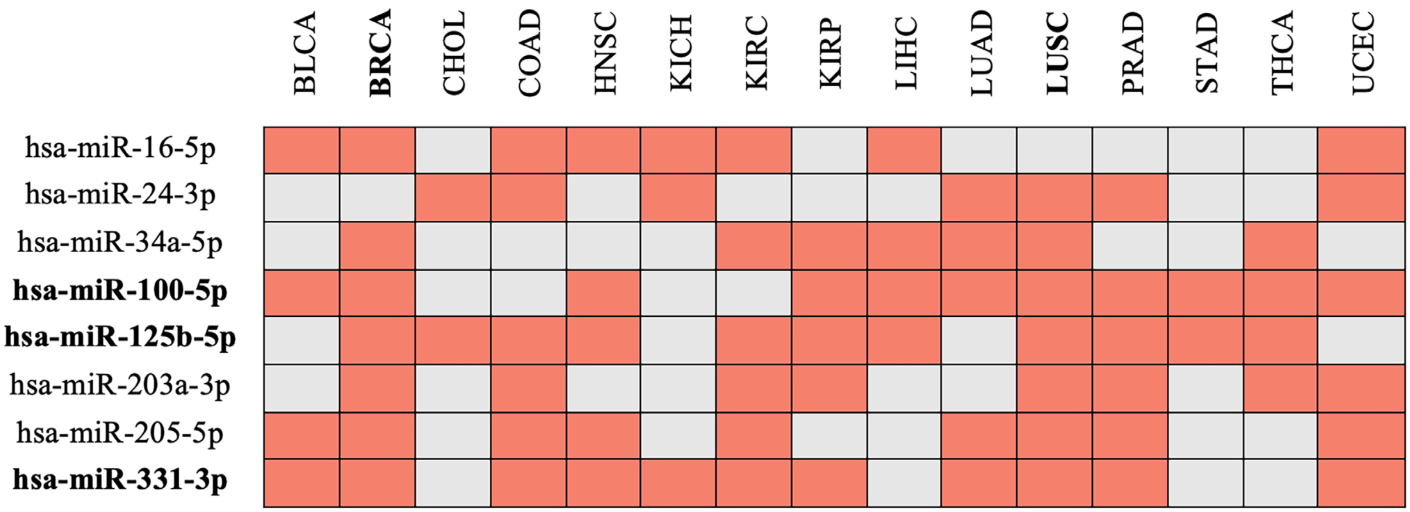

Supplementary Figure 1. Heatmap of statistically significant expression patterns of HPV-16-associated miRNAs across multiple TCGA datasets. Red cells indicate significant differential expression ($P < .001$), while gray cells represent non-significant results ($p \geq 0.001$). The highlighted miRNAs or cancer types in bold represent the most significant. (BLCA: Bladder Urothelial Carcinoma; BRCA: Breast Invasive Carcinoma; CHOL: Cholangiocarcinoma; COAD: Colon Adenocarcinoma; HNSC: Head and Neck Squamous Cell Carcinoma; KICH: Kidney Chromophobe; KIRC: Kidney Renal Clear Cell Carcinoma; KIRP: Kidney Renal Papillary Cell Carcinoma; LIHC: Liver Hepatocellular Carcinoma; LUAD: Lung Adenocarcinoma; LUSC: Lung Squamous Cell Carcinoma; PRAD: Prostate Adenocarcinoma; STAD: Stomach Adenocarcinoma; THCA: Thyroid Carcinoma; UCEC: Uterine Corpus Endometrial Carcinoma).

Supplementary Table 2. List of the predicted target genes, associated miRNAs, and prediction scores (score≥90)

Target Score	miRNA	Gene Symbol
100	hsa-miR-24-3p	BOK
100	hsa-miR-16-5p	DESI1
100	hsa-miR-34a-5p	DLL1
100	hsa-miR-34a-5p	FAM167A
100	hsa-miR-34a-5p	FAM76A
100	hsa-miR-16-5p	FASN
100	hsa-miR-16-5p	FGF2
100	hsa-miR-34a-5p	HCN3
100	hsa-miR-34a-5p	MDM4
100	hsa-miR-205-5p	MOSMO
100	hsa-miR-16-5p	PAPPA
100	hsa-miR-16-5p	PHF19
100	hsa-miR-16-5p	PTPN4
100	hsa-miR-34a-5p	RAP1GDS1
100	hsa-miR-34a-5p	SDK2
100	hsa-miR-16-5p	TNRC6B
100	hsa-miR-16-5p	UNC80
99	hsa-miR-203a-3p	ADAMTS6
99	hsa-miR-203a-3p	AFF4
99	hsa-miR-16-5p	ANKUB1
99	hsa-miR-24-3p	APBA1
99	hsa-miR-125b-5p	ARID3B
99	hsa-miR-16-5p	ARIH1
99	hsa-miR-16-5p	ARL2
99	hsa-miR-16-5p	ATG14
99	hsa-miR-24-3p	AVL9
99	hsa-miR-203a-3p	BBX
99	hsa-miR-205-5p	BICC1
99	hsa-miR-125b-5p	BMF
99	hsa-miR-16-5p	BTRC
99	hsa-miR-203a-3p	CAB39
99	hsa-miR-16-5p	CASK
99	hsa-miR-203a-3p	CASK
99	hsa-miR-16-5p	CCNE1
99	hsa-miR-205-5p	CDK19
99	hsa-miR-205-5p	CHN1
99	hsa-miR-24-3p	CHST4
99	hsa-miR-16-5p	CYB561A3
99	hsa-miR-16-5p	DCLK1
99	hsa-miR-203a-3p	DGKH
99	hsa-miR-34a-5p	E2F5
99	hsa-miR-24-3p	ELL
99	hsa-miR-203a-3p	ELL2

Supplementary Table 2. List of the predicted target genes, associated miRNAs, and prediction scores (score≥90) (*Continued*)

Target Score	miRNA	Gene Symbol
99	hsa-miR-16-5p	EPHB2
99	hsa-miR-16-5p	FBXO21
99	hsa-miR-16-5p	FGF7
99	hsa-miR-34a-5p	FKBP1B
99	hsa-miR-125b-5p	FREM1
99	hsa-miR-16-5p	GAREM1
99	hsa-miR-125b-5p	GCNT1
99	hsa-miR-203a-3p	GUCY1A2
99	hsa-miR-24-3p	IFFO2
99	hsa-miR-16-5p	LSM11
99	hsa-miR-16-5p	LUZP1
99	hsa-miR-203a-3p	MEX3C
99	hsa-miR-34a-5p	MGAT4A
99	hsa-miR-16-5p	MGAT4A
99	hsa-miR-34a-5p	NAV3
99	hsa-miR-16-5p	NECTIN1
99	hsa-miR-331-3p	NRP2
99	hsa-miR-16-5p	NUP50
99	hsa-miR-203a-3p	PDE4D
99	hsa-miR-203a-3p	PHIP
99	hsa-miR-34a-5p	PPP1R11
99	hsa-miR-205-5p	PPP2R2D
99	hsa-miR-125b-5p	PPP4R3A
99	hsa-miR-205-5p	RAB11FIP1
99	hsa-miR-34a-5p	SCN2B
99	hsa-miR-203a-3p	SEMA5A
99	hsa-miR-125b-5p	SH3TC2
99	hsa-miR-16-5p	SLC13A3
99	hsa-miR-16-5p	SPRYD3
99	hsa-miR-125b-5p	STARD13
99	hsa-miR-24-3p	STRADB
99	hsa-miR-34a-5p	SYT1
99	hsa-miR-24-3p	TAOK1
99	hsa-miR-203a-3p	THSD7A
99	hsa-miR-203a-3p	TMEM154
99	hsa-miR-16-5p	UBE2Q1
99	hsa-miR-16-5p	ZBTB46
99	hsa-miR-125b-5p	ZNF704
98	hsa-miR-205-5p	AAK1
98	hsa-miR-16-5p	ABL2
98	hsa-miR-16-5p	AHCYL2
98	hsa-miR-16-5p	AKT3

(*Continued*)

Supplementary Table 2. List of the predicted target genes, associated miRNAs, and prediction scores (score≥90) (*Continued*)

Target Score	miRNA	Gene Symbol
98	hsa-miR-203a-3p	ALG5
98	hsa-miR-16-5p	ANO3
98	hsa-miR-16-5p	APLN
98	hsa-miR-16-5p	ARL3
98	hsa-miR-16-5p	ATG9A
98	hsa-miR-16-5p	AXIN2
98	hsa-miR-203a-3p	B3GNT5
98	hsa-miR-24-3p	BCL2L11
98	hsa-miR-125b-5p	BLZF1
98	hsa-miR-205-5p	BTBD3
98	hsa-miR-16-5p	C2orf42
98	hsa-miR-34a-5p	CACNA1E
98	hsa-miR-16-5p	CACNA1E
98	hsa-miR-16-5p	CCND2
98	hsa-miR-34a-5p	CELF3
98	hsa-miR-24-3p	CMTM4
98	hsa-miR-16-5p	CPEB2
98	hsa-miR-203a-3p	DDX6
98	hsa-miR-203a-3p	FAM126B
98	hsa-miR-34a-5p	FLOT2
98	hsa-miR-24-3p	GBX2
98	hsa-miR-16-5p	GPR63
98	hsa-miR-203a-3p	HNRNPR
98	hsa-miR-203a-3p	ID4
98	hsa-miR-125b-5p	IER3IP1
98	hsa-miR-16-5p	IPO7
98	hsa-miR-16-5p	KCNJ2
98	hsa-miR-16-5p	KIF1B
98	hsa-miR-16-5p	KIF5C
98	hsa-miR-34a-5p	LGR4
98	hsa-miR-205-5p	LPCAT1
98	hsa-miR-203a-3p	MEF2C
98	hsa-miR-34a-5p	MET
98	hsa-miR-16-5p	MOB3B
98	hsa-miR-203a-3p	MORF4L1
98	hsa-miR-16-5p	MTMR3
98	hsa-miR-16-5p	MYB
98	hsa-miR-34a-5p	MYCN
98	hsa-miR-34a-5p	NAV1
98	hsa-miR-34a-5p	NECTIN1
98	hsa-miR-34a-5p	PACS1
98	hsa-miR-16-5p	PISD

(*Continued*)

Supplementary Table 2. List of the predicted target genes, associated miRNAs, and prediction scores (score≥90) (*Continued*)

Target Score	miRNA	Gene Symbol
98	hsa-miR-16-5p	*PLAG1*
98	hsa-miR-16-5p	*RASGEF1B*
98	hsa-miR-205-5p	*RBM47*
98	hsa-miR-16-5p	*RECK*
98	hsa-miR-203a-3p	*RP2*
98	hsa-miR-34a-5p	*SATB2*
98	hsa-miR-16-5p	*SCN8A*
98	hsa-miR-16-5p	*SLC11A2*
98	hsa-miR-16-5p	*SLC9A6*
98	hsa-miR-16-5p	*SPRED1*
98	hsa-miR-16-5p	*SREK1*
98	hsa-miR-16-5p	*SYNJ1*
98	hsa-miR-16-5p	*TBL1XR1*
98	hsa-miR-34a-5p	*TGIF2*
98	hsa-miR-16-5p	*TLK1*
98	hsa-miR-16-5p	*UBE2V1*
98	hsa-miR-16-5p	*UBE4B*
98	hsa-miR-16-5p	*UBN2*
98	hsa-miR-24-3p	*VSTM4*
98	hsa-miR-34a-5p	*XYLT1*
98	hsa-miR-16-5p	*ZBTB44*
97	hsa-miR-34a-5p	*ABR*
97	hsa-miR-125b-5p	*ACHE*
97	hsa-miR-34a-5p	*AHCYL2*
97	hsa-miR-24-3p	*AMOTL2*
97	hsa-miR-203a-3p	*AQP4*
97	hsa-miR-125b-5p	*BAK1*
97	hsa-miR-24-3p	*C8orf58*
97	hsa-miR-205-5p	*C9orf153*
97	hsa-miR-205-5p	*CCNJ*
97	hsa-miR-16-5p	*CDCA4*
97	hsa-miR-125b-5p	*CGN*
97	hsa-miR-203a-3p	*CLOCK*
97	hsa-miR-16-5p	*CYP26B1*
97	hsa-miR-16-5p	*DMPK*
97	hsa-miR-16-5p	*DNAJB4*
97	hsa-miR-125b-5p	*DOCK3*
97	hsa-miR-205-5p	*DSC2*
97	hsa-miR-24-3p	*FAM45A*
97	hsa-miR-16-5p	*FBXW7*
97	hsa-miR-34a-5p	*FUT9*
97	hsa-miR-16-5p	*GRM7*

(*Continued*)

Supplementary Table 2. List of the predicted target genes, associated miRNAs, and prediction scores (score≥90) (*Continued*)

Target Score	miRNA	Gene Symbol
97	hsa-miR-16-5p	*HTR2A*
97	hsa-miR-125b-5p	*IRF4*
97	hsa-miR-125b-5p	*KCNK10*
97	hsa-miR-125b-5p	*KCNS3*
97	hsa-miR-16-5p	*KDSR*
97	hsa-miR-203a-3p	*KIAA1211*
97	hsa-miR-16-5p	*KIF23*
97	hsa-miR-125b-5p	*KLF13*
97	hsa-miR-24-3p	*LRPAP1*
97	hsa-miR-16-5p	*LURAP1L*
97	hsa-miR-16-5p	*MAMSTR*
97	hsa-miR-205-5p	*MAP3K13*
97	hsa-miR-34a-5p	*MPP2*
97	hsa-miR-125b-5p	*NBEAL2*
97	hsa-miR-205-5p	*NFAT5*
97	hsa-miR-125b-5p	*NIPAL4*
97	hsa-miR-203a-3p	*NUFIP2*
97	hsa-miR-125b-5p	*NUP210*
97	hsa-miR-203a-3p	*OLFM3*
97	hsa-miR-34a-5p	*PKP4*
97	hsa-miR-203a-3p	*PLPP3*
97	hsa-miR-16-5p	*PPM1E*
97	hsa-miR-203a-3p	*PRPS2*
97	hsa-miR-24-3p	*PTGER4*
97	hsa-miR-203a-3p	*PTP4A1*
97	hsa-miR-205-5p	*PTPRJ*
97	hsa-miR-16-5p	*RAB11FIP2*
97	hsa-miR-203a-3p	*RAB27B*
97	hsa-miR-16-5p	*RASSF8*
97	hsa-miR-125b-5p	*RORA*
97	hsa-miR-34a-5p	*RRAS*
97	hsa-miR-24-3p	*SESN1*
97	hsa-miR-34a-5p	*SHANK3*
97	hsa-miR-16-5p	*SHOC2*
97	hsa-miR-16-5p	*STOX2*
97	hsa-miR-205-5p	*TAPT1*
97	hsa-miR-16-5p	*TFAP2A*
97	hsa-miR-16-5p	*TMEM100*
97	hsa-miR-125b-5p	*TMEM135*
97	hsa-miR-24-3p	*TOP1*
97	hsa-miR-203a-3p	*UBR1*
97	hsa-miR-34a-5p	*VAMP2*

(*Continued*)

Supplementary Table 2. List of the predicted target genes, associated miRNAs, and prediction scores (score≥90) (*Continued*)

Target Score	miRNA	Gene Symbol
97	hsa-miR-203a-3p	*VSNL1*
97	hsa-miR-16-5p	*WEE1*
97	hsa-miR-24-3p	*ZXDB*
96	hsa-miR-16-5p	*ARMH4*
96	hsa-miR-205-5p	*C11orf86*
96	hsa-miR-205-5p	*CADM1*
96	hsa-miR-205-5p	*CALCRL*
96	hsa-miR-34a-5p	*CAMTA1*
96	hsa-miR-16-5p	*CASR*
96	hsa-miR-16-5p	*CBX2*
96	hsa-miR-16-5p	*CCDC6*
96	hsa-miR-205-5p	*CDH11*
96	hsa-miR-16-5p	*CFAP45*
96	hsa-miR-16-5p	*CNOT6L*
96	hsa-miR-205-5p	*CPSF6*
96	hsa-miR-34a-5p	*CUEDC1*
96	hsa-miR-125b-5p	*CYP24A1*
96	hsa-miR-24-3p	*DNAJB12*
96	hsa-miR-203a-3p	*DUSP5*
96	hsa-miR-16-5p	*EDA*
96	hsa-miR-203a-3p	*EGLN1*
96	hsa-miR-203a-3p	*EPYC*
96	hsa-miR-203a-3p	*ERAP1*
96	hsa-miR-24-3p	*FAM168B*
96	hsa-miR-16-5p	*GABARAPL1*
96	hsa-miR-203a-3p	*GRHL3*
96	hsa-miR-24-3p	*INSIG1*
96	hsa-miR-125b-5p	*JADE2*
96	hsa-miR-125b-5p	*KCTD15*
96	hsa-miR-24-3p	*KCTD21*
96	hsa-miR-16-5p	*KLHL2*
96	hsa-miR-203a-3p	*MAP3K1*
96	hsa-miR-203a-3p	*MAP3K13*
96	hsa-miR-24-3p	*MATR3*
96	hsa-miR-203a-3p	*MBNL3*
96	hsa-miR-205-5p	*MGRN1*
96	hsa-miR-34a-5p	*MLLT3*
96	hsa-miR-24-3p	*MPDU1*
96	hsa-miR-16-5p	*MYLK*
96	hsa-miR-16-5p	*N4BP1*
96	hsa-miR-16-5p	*NAPG*
96	hsa-miR-125b-5p	*NPL*

(*Continued*)

Supplementary Table 2. List of the predicted target genes, associated miRNAs, and prediction scores (score≥90) (*Continued*)

Target Score	miRNA	Gene Symbol
96	hsa-miR-203a-3p	*NUDT21*
96	hsa-miR-16-5p	*PAFAH1B1*
96	hsa-miR-16-5p	*PAFAH1B2*
96	hsa-miR-16-5p	*PCMT1*
96	hsa-miR-24-3p	*PER2*
96	hsa-miR-331-3p	*PHLPP1*
96	hsa-miR-125b-5p	*PI4K2B*
96	hsa-miR-24-3p	*PIM2*
96	hsa-miR-34a-5p	*PITPNC1*
96	hsa-miR-205-5p	*PLCB1*
96	hsa-miR-16-5p	*PLPP1*
96	hsa-miR-331-3p	*PTPN2*
96	hsa-miR-16-5p	*PTPN3*
96	hsa-miR-16-5p	*RFX3*
96	hsa-miR-24-3p	*RNF138*
96	hsa-miR-16-5p	*RNF144B*
96	hsa-miR-203a-3p	*RTKN2*
96	hsa-miR-16-5p	*SALL4*
96	hsa-miR-16-5p	*SEC24A*
96	hsa-miR-16-5p	*SEMA6D*
96	hsa-miR-34a-5p	*SLC25A27*
96	hsa-miR-203a-3p	*SNAI2*
96	hsa-miR-16-5p	*STXBP3*
96	hsa-miR-16-5p	*TBPL1*
96	hsa-miR-203a-3p	*TC2N*
96	hsa-miR-205-5p	*TNFAIP8*
96	hsa-miR-100-5p	*TRIB2*
96	hsa-miR-24-3p	*TRPC4AP*
96	hsa-miR-125b-5p	*TTPA*
96	hsa-miR-24-3p	*VGLL3*
96	hsa-miR-331-3p	*ZBTB2*
96	hsa-miR-203a-3p	*ZBTB20*
96	hsa-miR-16-5p	*ZBTB34*
96	hsa-miR-16-5p	*ZCCHC3*
96	hsa-miR-205-5p	*ZFYVE16*
96	hsa-miR-203a-3p	*ZNF148*
96	hsa-miR-203a-3p	*ZNF281*
96	hsa-miR-16-5p	*ZNF367*
96	hsa-miR-125b-5p	*ZNF543*
96	hsa-miR-205-5p	*ZNF606*
96	hsa-miR-16-5p	*ZNF691*
96	hsa-miR-125b-5p	*ZSCAN29*
96	hsa-miR-125b-5p	*ZSWIM6*
95	hsa-miR-125b-5p	*ANAPC16*
95	hsa-miR-205-5p	*CASC4*
95	hsa-miR-24-3p	*CASTOR2*
95	hsa-miR-16-5p	*CBX4*
95	hsa-miR-24-3p	*CCDC58*
95	hsa-miR-125b-5p	*CDH5*
95	hsa-miR-16-5p	*CEP55*
95	hsa-miR-16-5p	*CHAC1*
95	hsa-miR-24-3p	*CITED4*
95	hsa-miR-203a-3p	*CRISP1*
95	hsa-miR-34a-5p	*CYREN*
95	hsa-miR-125b-5p	*DUS1L*
95	hsa-miR-125b-5p	*ENPEP*
95	hsa-miR-125b-5p	*ETS1*
95	hsa-miR-16-5p	*FGFR1*
95	hsa-miR-203a-3p	*FGFR1OP*
95	hsa-miR-34a-5p	*FOXP1*
95	hsa-miR-34a-5p	*FUT8*
95	hsa-miR-16-5p	*GPATCH8*
95	hsa-miR-16-5p	*HIPK2*
95	hsa-miR-205-5p	*HS3ST1*
95	hsa-miR-203a-3p	*IKZF5*
95	hsa-miR-203a-3p	*IMPG1*
95	hsa-miR-16-5p	*IPPK*
95	hsa-miR-125b-5p	*KIAA1841*
95	hsa-miR-16-5p	*KIF5B*
95	hsa-miR-24-3p	*KLHL1*
95	hsa-miR-16-5p	*LATS1*
95	hsa-miR-205-5p	*LCOR*
95	hsa-miR-24-3p	*LIMD1*
95	hsa-miR-125b-5p	*LIPA*
95	hsa-miR-205-5p	*LRP6*
95	hsa-miR-203a-3p	*LRRTM2*
95	hsa-miR-205-5p	*MAGI2*
95	hsa-miR-16-5p	*MAP2K1*
95	hsa-miR-34a-5p	*MEX3C*
95	hsa-miR-203a-3p	*MICAL2*
95	hsa-miR-16-5p	*MKX*
95	hsa-miR-24-3p	*MLEC*
95	hsa-miR-203a-3p	*MORF4L2*
95	hsa-miR-125b-5p	*MTF1*
95	hsa-miR-16-5p	*MYBL1*
95	hsa-miR-203a-3p	*MYEF2*
95	hsa-miR-125b-5p	*NECAB3*
95	hsa-miR-205-5p	*NECAP1*
95	hsa-miR-24-3p	*NLK*
95	hsa-miR-34a-5p	*NPNT*
95	hsa-miR-125b-5p	*OSBPL9*
95	hsa-miR-203a-3p	*PDAP1*
95	hsa-miR-205-5p	*PRKCE*
95	hsa-miR-125b-5p	*PRTG*
95	hsa-miR-125b-5p	*SBNO1*
95	hsa-miR-16-5p	*SETD3*
95	hsa-miR-16-5p	*SLC25A37*
95	hsa-miR-205-5p	*SLC35B3*
95	hsa-miR-125b-5p	*SLC37A2*
95	hsa-miR-203a-3p	*SLC4A4*
95	hsa-miR-16-5p	*SMURF1*
95	hsa-miR-203a-3p	*SRA1*
95	hsa-miR-34a-5p	*SRPRA*
95	hsa-miR-125b-5p	*SSTR3*
95	hsa-miR-24-3p	*STC2*
95	hsa-miR-34a-5p	*TBL1XR1*
95	hsa-miR-16-5p	*TNFSF13B*
95	hsa-miR-34a-5p	*TOB2*
95	hsa-miR-203a-3p	*TPR*
95	hsa-miR-203a-3p	*TXNDC16*
95	hsa-miR-16-5p	*UBFD1*
95	hsa-miR-24-3p	*UCK1*
95	hsa-miR-16-5p	*USP25*
95	hsa-miR-203a-3p	*VAPA*
95	hsa-miR-16-5p	*VEGFA*
95	hsa-miR-125b-5p	*VPS4B*
95	hsa-miR-205-5p	*VTI1B*
95	hsa-miR-203a-3p	*ZBTB11*
95	hsa-miR-16-5p	*ZMAT3*
95	hsa-miR-203a-3p	*ZNF197*
95	hsa-miR-203a-3p	*ZNF680*
94	hsa-miR-24-3p	*ABCB9*
94	hsa-miR-125b-5p	*ABHD6*
94	hsa-miR-205-5p	*AMOT*
94	hsa-miR-203a-3p	*ARHGAP42*
94	hsa-miR-16-5p	*ARHGDIA*

(*Continued*)

Supplementary Table 2. List of the predicted target genes, associated miRNAs, and prediction scores (score≥90) (*Continued*)

Target Score	miRNA	Gene Symbol
94	hsa-miR-203a-3p	ATF2
94	hsa-miR-125b-5p	ATP10D
94	hsa-miR-125b-5p	ATXN1
94	hsa-miR-16-5p	ATXN2
94	hsa-miR-125b-5p	BAP1
94	hsa-miR-125b-5p	C19orf38
94	hsa-miR-203a-3p	C2orf80
94	hsa-miR-24-3p	CCDC157
94	hsa-miR-205-5p	CCDC80
94	hsa-miR-205-5p	CDK14
94	hsa-miR-16-5p	CHEK1
94	hsa-miR-203a-3p	CLSTN3
94	hsa-miR-205-5p	CPEB2
94	hsa-miR-331-3p	CPSF2
94	hsa-miR-16-5p	DCP1A
94	hsa-miR-16-5p	DDX3X
94	hsa-miR-205-5p	DUSP7
94	hsa-miR-24-3p	DYRK2
94	hsa-miR-34a-5p	ELMOD1
94	hsa-miR-205-5p	ERBB4
94	hsa-miR-205-5p	EVA1C
94	hsa-miR-34a-5p	FGD6
94	hsa-miR-34a-5p	FOXN2
94	hsa-miR-34a-5p	GABRA3
94	hsa-miR-16-5p	GALNT13
94	hsa-miR-205-5p	HERC3
94	hsa-miR-24-3p	IFNG
94	hsa-miR-34a-5p	JAKMIP1
94	hsa-miR-16-5p	JPH3
94	hsa-miR-203a-3p	KAT6A
94	hsa-miR-100-5p	KBTBD8
94	hsa-miR-125b-5p	KCNIP3
94	hsa-miR-34a-5p	KIAA1217
94	hsa-miR-203a-3p	KRT35
94	hsa-miR-125b-5p	LACTB
94	hsa-miR-34a-5p	LEF1
94	hsa-miR-24-3p	LMBR1L
94	hsa-miR-16-5p	LRIG2
94	hsa-miR-16-5p	LRRN3
94	hsa-miR-203a-3p	MAP1B
94	hsa-miR-125b-5p	MAPRE2
94	hsa-miR-24-3p	MBOAT7

(*Continued*)

Supplementary Table 2. List of the predicted target genes, associated miRNAs, and prediction scores (score≥90) (*Continued*)

Target Score	miRNA	Gene Symbol
94	hsa-miR-24-3p	MGAT4A
94	hsa-miR-24-3p	MIDN
94	hsa-miR-125b-5p	MORC2
94	hsa-miR-24-3p	NDST3
94	hsa-miR-24-3p	NRP2
94	hsa-miR-34a-5p	NUMBL
94	hsa-miR-16-5p	OMG
94	hsa-miR-16-5p	OOEP
94	hsa-miR-203a-3p	OSBPL8
94	hsa-miR-16-5p	P3H2
94	hsa-miR-205-5p	PDE3B
94	hsa-miR-24-3p	PLCL2
94	hsa-miR-16-5p	PLXNA4
94	hsa-miR-34a-5p	PPARGC1B
94	hsa-miR-125b-5p	PPIL2
94	hsa-miR-16-5p	PPP1R11
94	hsa-miR-16-5p	PPP2R1B
94	hsa-miR-205-5p	PTPRM
94	hsa-miR-16-5p	PTPRR
94	hsa-miR-205-5p	QKI
94	hsa-miR-24-3p	RANBP10
94	hsa-miR-125b-5p	RAPGEF5
94	hsa-miR-125b-5p	RBM20
94	hsa-miR-16-5p	RNF217
94	hsa-miR-205-5p	ROCK2
94	hsa-miR-24-3p	SCML2
94	hsa-miR-203a-3p	SCYL2
94	hsa-miR-125b-5p	SEL1L
94	hsa-miR-125b-5p	SEMA4B
94	hsa-miR-205-5p	SLC19A2
94	hsa-miR-125b-5p	SLC35A4
94	hsa-miR-125b-5p	SLC39A9
94	hsa-miR-100-5p	SMARCA5
94	hsa-miR-34a-5p	SNAI1
94	hsa-miR-205-5p	SPANXN5
94	hsa-miR-16-5p	SRPRA
94	hsa-miR-16-5p	STRADB
94	hsa-miR-16-5p	STXBP5
94	hsa-miR-125b-5p	SYDE2
94	hsa-miR-34a-5p	TMEM255A
94	hsa-miR-203a-3p	TMPRSS11D
94	hsa-miR-34a-5p	TNRC18

(*Continued*)

Supplementary Table 2. List of the predicted target genes, associated miRNAs, and prediction scores (score≥90) (*Continued*)

Target Score	miRNA	Gene Symbol
94	hsa-miR-34a-5p	TPPP
94	hsa-miR-24-3p	TTC17
94	hsa-miR-34a-5p	UBP1
94	hsa-miR-125b-5p	UCK2
94	hsa-miR-24-3p	UGCG
94	hsa-miR-16-5p	USP42
94	hsa-miR-24-3p	VANGL2
94	hsa-miR-203a-3p	VIRMA
94	hsa-miR-16-5p	WNK3
94	hsa-miR-203a-3p	YTHDF3
94	hsa-miR-16-5p	ZBTB39
94	hsa-miR-16-5p	ZFHX4
94	hsa-miR-16-5p	ZNF622
94	hsa-miR-331-3p	ZNF652
94	hsa-miR-16-5p	ZNRF2
94	hsa-miR-24-3p	ZXDA
93	hsa-miR-203a-3p	ACSL1
93	hsa-miR-16-5p	ACTR2
93	hsa-miR-24-3p	ACVR1B
93	hsa-miR-205-5p	AFDN
93	hsa-miR-16-5p	AMOTL1
93	hsa-miR-24-3p	ANKRD44
93	hsa-miR-205-5p	ANKRD50
93	hsa-miR-203a-3p	ARMT1
93	hsa-miR-34a-5p	ASIC2
93	hsa-miR-24-3p	ATAD2B
93	hsa-miR-16-5p	AVL9
93	hsa-miR-203a-3p	BCCIP
93	hsa-miR-34a-5p	BMP3
93	hsa-miR-16-5p	BTAF1
93	hsa-miR-24-3p	C2orf72
93	hsa-miR-24-3p	CAMK2B
93	hsa-miR-16-5p	CCND1
93	hsa-miR-125b-5p	CCNJ
93	hsa-miR-16-5p	CD2AP
93	hsa-miR-24-3p	CDH7
93	hsa-miR-24-3p	CDV3
93	hsa-miR-34a-5p	CERS6
93	hsa-miR-203a-3p	COL17A1
93	hsa-miR-34a-5p	DAAM1
93	hsa-miR-331-3p	DCLRE1B
93	hsa-miR-125b-5p	DHX33

(*Continued*)

Supplementary Table 2. List of the predicted target genes, associated miRNAs, and prediction scores (score≥90) (*Continued*)

Target Score	miRNA	Gene Symbol
93	hsa-miR-16-5p	*DIXDC1*
93	hsa-miR-203a-3p	*DLG5*
93	hsa-miR-205-5p	*DMXL2*
93	hsa-miR-34a-5p	*DNM1L*
93	hsa-miR-34a-5p	*EML5*
93	hsa-miR-205-5p	*ERRFI1*
93	hsa-miR-205-5p	*ETNK1*
93	hsa-miR-205-5p	*EZR*
93	hsa-miR-203a-3p	*FAM84A*
93	hsa-miR-16-5p	*FBXL20*
93	hsa-miR-125b-5p	*FIBP*
93	hsa-miR-205-5p	*FOXF1*
93	hsa-miR-34a-5p	*FOXJ2*
93	hsa-miR-16-5p	*FOXK1*
93	hsa-miR-24-3p	*FST*
93	hsa-miR-34a-5p	*GALNT7*
93	hsa-miR-24-3p	*GCNA*
93	hsa-miR-16-5p	*GHR*
93	hsa-miR-205-5p	*GPC6*
93	hsa-miR-34a-5p	*GPR22*
93	hsa-miR-24-3p	*HNF1B*
93	hsa-miR-34a-5p	*HNF4A*
93	hsa-miR-16-5p	*HSPA4L*
93	hsa-miR-16-5p	*HSPG2*
93	hsa-miR-16-5p	*KANK1*
93	hsa-miR-125b-5p	*KIAA1522*
93	hsa-miR-16-5p	*KIF21A*
93	hsa-miR-24-3p	*LPAR6*
93	hsa-miR-203a-3p	*LRCH2*
93	hsa-miR-205-5p	*LRRK2*
93	hsa-miR-203a-3p	*MACO1*
93	hsa-miR-16-5p	*MFN2*
93	hsa-miR-24-3p	*MIA3*
93	hsa-miR-203a-3p	*MID1*
93	hsa-miR-100-5p	*MTOR*
93	hsa-miR-16-5p	*MYO5A*
93	hsa-miR-205-5p	*NFIB*
93	hsa-miR-205-5p	*NKD1*
93	hsa-miR-125b-5p	*OLFML2A*
93	hsa-miR-34a-5p	*OLIG3*
93	hsa-miR-24-3p	*PAQR3*
93	hsa-miR-125b-5p	*PDZD3*

(*Continued*)

Supplementary Table 2. List of the predicted target genes, associated miRNAs, and prediction scores (score≥90) (*Continued*)

Target Score	miRNA	Gene Symbol
93	hsa-miR-203a-3p	*PHF12*
93	hsa-miR-125b-5p	*PHF20*
93	hsa-miR-203a-3p	*PHF6*
93	hsa-miR-203a-3p	*PHLDA1*
93	hsa-miR-203a-3p	*PIK3CA*
93	hsa-miR-16-5p	*PLXNC1*
93	hsa-miR-34a-5p	*PPFIA1*
93	hsa-miR-203a-3p	*PRKAG2*
93	hsa-miR-203a-3p	*PRKG1*
93	hsa-miR-203a-3p	*PTPN4*
93	hsa-miR-24-3p	*PTPRD*
93	hsa-miR-24-3p	*RALA*
93	hsa-miR-24-3p	*RASA1*
93	hsa-miR-125b-5p	*RASGRF2*
93	hsa-miR-16-5p	*RBPJ*
93	hsa-miR-16-5p	*RPS6KA3*
93	hsa-miR-34a-5p	*RPS6KL1*
93	hsa-miR-205-5p	*RTN3*
93	hsa-miR-203a-3p	*SCP2*
93	hsa-miR-16-5p	*SEL1L3*
93	hsa-miR-125b-5p	*SGPL1*
93	hsa-miR-24-3p	*SMAGP*
93	hsa-miR-24-3p	*SNN*
93	hsa-miR-16-5p	*SOCS6*
93	hsa-miR-203a-3p	*SPOCK3*
93	hsa-miR-24-3p	*SSR1*
93	hsa-miR-205-5p	*STRBP*
93	hsa-miR-16-5p	*SUCO*
93	hsa-miR-16-5p	*SYDE2*
93	hsa-miR-16-5p	*SYT3*
93	hsa-miR-203a-3p	*TARDBP*
93	hsa-miR-34a-5p	*TASOR*
93	hsa-miR-24-3p	*TLN2*
93	hsa-miR-125b-5p	*TMEM120B*
93	hsa-miR-24-3p	*TMEM161B*
93	hsa-miR-125b-5p	*TMEM168*
93	hsa-miR-16-5p	*TMEM245*
93	hsa-miR-24-3p	*TMEM50B*
93	hsa-miR-24-3p	*TOR2A*
93	hsa-miR-16-5p	*TRANK1*
93	hsa-miR-331-3p	*TSPAN18*
93	hsa-miR-16-5p	*UBE4A*

(*Continued*)

Supplementary Table 2. List of the predicted target genes, associated miRNAs, and prediction scores (score≥90) (*Continued*)

Target Score	miRNA	Gene Symbol
93	hsa-miR-125b-5p	*UBN1*
93	hsa-miR-16-5p	*VPS33B*
93	hsa-miR-125b-5p	*VTCN1*
93	hsa-miR-16-5p	*WNT3A*
93	hsa-miR-125b-5p	*XKRX*
93	hsa-miR-24-3p	*ZCCHC14*
93	hsa-miR-34a-5p	*ZMYM4*
93	hsa-miR-24-3p	*ZNF572*
92	hsa-miR-203a-3p	*ABCE1*
92	hsa-miR-125b-5p	*ABHD3*
92	hsa-miR-125b-5p	*ACER2*
92	hsa-miR-16-5p	*ACVR2A*
92	hsa-miR-203a-3p	*ADAM12*
92	hsa-miR-16-5p	*ADAMTS3*
92	hsa-miR-24-3p	*ADD2*
92	hsa-miR-34a-5p	*ADO*
92	hsa-miR-16-5p	*AGO4*
92	hsa-miR-203a-3p	*AKAP7*
92	hsa-miR-34a-5p	*AKIP1*
92	hsa-miR-125b-5p	*ALPK3*
92	hsa-miR-203a-3p	*ANKRD52*
92	hsa-miR-34a-5p	*ARID4B*
92	hsa-miR-16-5p	*ASH1L*
92	hsa-miR-34a-5p	*ATMIN*
92	hsa-miR-16-5p	*ATXN7L2*
92	hsa-miR-331-3p	*BAIAP2*
92	hsa-miR-24-3p	*BTN2A2*
92	hsa-miR-203a-3p	*C11orf91*
92	hsa-miR-24-3p	*C17orf78*
92	hsa-miR-205-5p	*C6orf222*
92	hsa-miR-34a-5p	*CACNB3*
92	hsa-miR-16-5p	*CACUL1*
92	hsa-miR-203a-3p	*CADM2*
92	hsa-miR-24-3p	*CALCR*
92	hsa-miR-203a-3p	*CCDC50*
92	hsa-miR-16-5p	*CD47*
92	hsa-miR-16-5p	*CDC25A*
92	hsa-miR-34a-5p	*CDK6*
92	hsa-miR-16-5p	*CHD2*
92	hsa-miR-205-5p	*CLDN11*
92	hsa-miR-205-5p	*CLTC*
92	hsa-miR-16-5p	*COP1*

(*Continued*)

Supplementary Table 2. List of the predicted target genes, associated miRNAs, and prediction scores (score≥90) (*Continued*)

Target Score	miRNA	Gene Symbol
92	hsa-miR-16-5p	*CPEB3*
92	hsa-miR-34a-5p	*CREB3L2*
92	hsa-miR-203a-3p	*CSN2*
92	hsa-miR-203a-3p	*DENND6A*
92	hsa-miR-125b-5p	*DIS3L2*
92	hsa-miR-16-5p	*DLL1*
92	hsa-miR-203a-3p	*DLX5*
92	hsa-miR-16-5p	*E2F3*
92	hsa-miR-203a-3p	*E2F3*
92	hsa-miR-24-3p	*EDA2R*
92	hsa-miR-203a-3p	*EGR3*
92	hsa-miR-34a-5p	*FAM117B*
92	hsa-miR-203a-3p	*FAM92A*
92	hsa-miR-203a-3p	*FAT3*
92	hsa-miR-16-5p	*G2E3*
92	hsa-miR-205-5p	*GABRA4*
92	hsa-miR-24-3p	*GAD1*
92	hsa-miR-34a-5p	*GOLPH3L*
92	hsa-miR-24-3p	*GRIP1*
92	hsa-miR-203a-3p	*GSKIP*
92	hsa-miR-205-5p	*HSD17B11*
92	hsa-miR-205-5p	*HSF5*
92	hsa-miR-16-5p	*IFT74*
92	hsa-miR-34a-5p	*IL6R*
92	hsa-miR-125b-5p	*INO80D*
92	hsa-miR-205-5p	*INPP4A*
92	hsa-miR-16-5p	*KCNK10*
92	hsa-miR-34a-5p	*KDM5D*
92	hsa-miR-24-3p	*KLHL3*
92	hsa-miR-16-5p	*KRTAP11-1*
92	hsa-miR-16-5p	*LAMP3*
92	hsa-miR-203a-3p	*LIN7A*
92	hsa-miR-16-5p	*LITAF*
92	hsa-miR-34a-5p	*LMAN1*
92	hsa-miR-205-5p	*LRP1*
92	hsa-miR-205-5p	*MARCKS*
92	hsa-miR-16-5p	*MEOX2*
92	hsa-miR-16-5p	*MEX3C*
92	hsa-miR-203a-3p	*MINDY2*
92	hsa-miR-205-5p	*MSI2*
92	hsa-miR-24-3p	*MTHFR*
92	hsa-miR-16-5p	*NAA25*

(*Continued*)

Supplementary Table 2. List of the predicted target genes, associated miRNAs, and prediction scores (score≥90) (*Continued*)

Target Score	miRNA	Gene Symbol
92	hsa-miR-203a-3p	*NAA30*
92	hsa-miR-125b-5p	*NCAN*
92	hsa-miR-24-3p	*NCOA5*
92	hsa-miR-16-5p	*NHLRC2*
92	hsa-miR-203a-3p	*NLK*
92	hsa-miR-34a-5p	*NRN1*
92	hsa-miR-205-5p	*P2RY1*
92	hsa-miR-16-5p	*PCDH17*
92	hsa-miR-16-5p	*PIAS2*
92	hsa-miR-203a-3p	*PLD1*
92	hsa-miR-125b-5p	*PPME1*
92	hsa-miR-125b-5p	*PRDM1*
92	hsa-miR-16-5p	*PTH*
92	hsa-miR-125b-5p	*RABEP2*
92	hsa-miR-125b-5p	*RABL6*
92	hsa-miR-16-5p	*RAD23B*
92	hsa-miR-203a-3p	*RAPGEF1*
92	hsa-miR-125b-5p	*REST*
92	hsa-miR-16-5p	*RETREG2*
92	hsa-miR-203a-3p	*RFX7*
92	hsa-miR-203a-3p	*RICTOR*
92	hsa-miR-16-5p	*RNF10*
92	hsa-miR-24-3p	*RNF2*
92	hsa-miR-205-5p	*RORA*
92	hsa-miR-125b-5p	*SARM1*
92	hsa-miR-205-5p	*SATB2*
92	hsa-miR-331-3p	*SEMA7A*
92	hsa-miR-205-5p	*SEPT4*
92	hsa-miR-34a-5p	*SERPINF2*
92	hsa-miR-205-5p	*SGMS1*
92	hsa-miR-24-3p	*SH2B3*
92	hsa-miR-205-5p	*SIPA1L1*
92	hsa-miR-16-5p	*SIRT4*
92	hsa-miR-331-3p	*SLAMF9*
92	hsa-miR-16-5p	*SLC12A2*
92	hsa-miR-16-5p	*SLC20A2*
92	hsa-miR-125b-5p	*SLC25A15*
92	hsa-miR-125b-5p	*SLC25A35*
92	hsa-miR-205-5p	*SLC35A1*
92	hsa-miR-34a-5p	*SLC4A7*
92	hsa-miR-125b-5p	*SLC7A1*
92	hsa-miR-16-5p	*SLIT2*

(*Continued*)

Supplementary Table 2. List of the predicted target genes, associated miRNAs, and prediction scores (score≥90) (*Continued*)

Target Score	miRNA	Gene Symbol
92	hsa-miR-16-5p	*SMAD7*
92	hsa-miR-24-3p	*ST3GAL1*
92	hsa-miR-16-5p	*STK33*
92	hsa-miR-34a-5p	*STRN3*
92	hsa-miR-16-5p	*SUMO3*
92	hsa-miR-34a-5p	*TAF4B*
92	hsa-miR-125b-5p	*TAZ*
92	hsa-miR-16-5p	*TBP*
92	hsa-miR-203a-3p	*TCF4*
92	hsa-miR-16-5p	*TMCC1*
92	hsa-miR-16-5p	*TMEM178B*
92	hsa-miR-16-5p	*TMEM183A*
92	hsa-miR-34a-5p	*TMEM184B*
92	hsa-miR-16-5p	*TRABD2B*
92	hsa-miR-125b-5p	*TRIM71*
92	hsa-miR-24-3p	*TSPAN14*
92	hsa-miR-203a-3p	*UBE2D3*
92	hsa-miR-34a-5p	*UCN2*
92	hsa-miR-34a-5p	*UNC13C*
92	hsa-miR-24-3p	*USF3*
92	hsa-miR-125b-5p	*USP2*
92	hsa-miR-24-3p	*VCPIP1*
92	hsa-miR-125b-5p	*WARS*
92	hsa-miR-203a-3p	*WDFY3*
92	hsa-miR-203a-3p	*WFDC13*
92	hsa-miR-16-5p	*XPO7*
92	hsa-miR-16-5p	*YTHDC1*
92	hsa-miR-16-5p	*ZMYM2*
92	hsa-miR-24-3p	*ZNF217*
92	hsa-miR-16-5p	*ZNF449*
92	hsa-miR-125b-5p	*ZSWIM5*
91	hsa-miR-203a-3p	*ADAMTS5*
91	hsa-miR-205-5p	*ADAMTS9*
91	hsa-miR-34a-5p	*AMER1*
91	hsa-miR-16-5p	*AMER1*
91	hsa-miR-34a-5p	*ANK3*
91	hsa-miR-203a-3p	*ATP2C1*
91	hsa-miR-16-5p	*ATXN7L1*
91	hsa-miR-24-3p	*B4GAT1*
91	hsa-miR-16-5p	*CC2D1B*
91	hsa-miR-16-5p	*CCNT1*
91	hsa-miR-24-3p	*CD28*

(*Continued*)

Supplementary Table 2. List of the predicted target genes, associated miRNAs, and prediction scores (score≥90) (*Continued*)

Target Score	miRNA	Gene Symbol
91	hsa-miR-16-5p	*CDC42SE2*
91	hsa-miR-24-3p	*CDK17*
91	hsa-miR-203a-3p	*CFAP97*
91	hsa-miR-16-5p	*CLOCK*
91	hsa-miR-331-3p	*CNTNAP4*
91	hsa-miR-16-5p	*CSDE1*
91	hsa-miR-34a-5p	*CTNND2*
91	hsa-miR-125b-5p	*DIRAS1*
91	hsa-miR-125b-5p	*DPH2*
91	hsa-miR-125b-5p	*DTX4*
91	hsa-miR-203a-3p	*DUS1L*
91	hsa-miR-205-5p	*E2F5*
91	hsa-miR-205-5p	*ERBB3*
91	hsa-miR-16-5p	*EZH1*
91	hsa-miR-24-3p	*FAM78B*
91	hsa-miR-34a-5p	*FGF23*
91	hsa-miR-125b-5p	*FUT4*
91	hsa-miR-205-5p	*FZD3*
91	hsa-miR-203a-3p	*GABRA1*
91	hsa-miR-16-5p	*GATAD2A*
91	hsa-miR-203a-3p	*GXYLT1*
91	hsa-miR-100-5p	*HS3ST2*
91	hsa-miR-34a-5p	*HSPA1B*
91	hsa-miR-125b-5p	*IL31*
91	hsa-miR-16-5p	*INSR*
91	hsa-miR-203a-3p	*INTS14*
91	hsa-miR-125b-5p	*INTS7*
91	hsa-miR-24-3p	*KCNB1*
91	hsa-miR-24-3p	*KCNK2*
91	hsa-miR-203a-3p	*KIF2A*
91	hsa-miR-203a-3p	*KLHL4*
91	hsa-miR-205-5p	*KPNA1*
91	hsa-miR-203a-3p	*KRT85*
91	hsa-miR-16-5p	*KRTAP4-6*
91	hsa-miR-205-5p	*LAMC1*
91	hsa-miR-16-5p	*LARGE2*
91	hsa-miR-203a-3p	*LIFR*
91	hsa-miR-24-3p	*LIMD2*
91	hsa-miR-16-5p	*LRRK1*
91	hsa-miR-203a-3p	*NOCT*
91	hsa-miR-16-5p	*NOS1*
91	hsa-miR-125b-5p	*NRXN1*

(*Continued*)

Supplementary Table 2. List of the predicted target genes, associated miRNAs, and prediction scores (score≥90) (*Continued*)

Target Score	miRNA	Gene Symbol
91	hsa-miR-125b-5p	*PAFAH1B1*
91	hsa-miR-203a-3p	*PAQR3*
91	hsa-miR-205-5p	*PAX9*
91	hsa-miR-34a-5p	*PDE7B*
91	hsa-miR-16-5p	*POU2F1*
91	hsa-miR-16-5p	*PPM1A*
91	hsa-miR-34a-5p	*PPP2R3A*
91	hsa-miR-24-3p	*PRKCH*
91	hsa-miR-205-5p	*PSD3*
91	hsa-miR-203a-3p	*PSD3*
91	hsa-miR-34a-5p	*PURB*
91	hsa-miR-203a-3p	*PURG*
91	hsa-miR-16-5p	*RAB9B*
91	hsa-miR-16-5p	*RARB*
91	hsa-miR-125b-5p	*RASGRF1*
91	hsa-miR-100-5p	*RAVER2*
91	hsa-miR-205-5p	*RCBTB1*
91	hsa-miR-24-3p	*RNF165*
91	hsa-miR-34a-5p	*RPGRIP1L*
91	hsa-miR-16-5p	*RPS6KA6*
91	hsa-miR-24-3p	*RUBCN*
91	hsa-miR-16-5p	*SALL1*
91	hsa-miR-203a-3p	*SCGB2A1*
91	hsa-miR-16-5p	*SIK1*
91	hsa-miR-125b-5p	*SLITRK6*
91	hsa-miR-34a-5p	*SMIM15*
91	hsa-miR-203a-3p	*SOX5*
91	hsa-miR-203a-3p	*SPARC*
91	hsa-miR-16-5p	*SPTLC1*
91	hsa-miR-24-3p	*SSTR1*
91	hsa-miR-125b-5p	*SYVN1*
91	hsa-miR-16-5p	*TLL1*
91	hsa-miR-125b-5p	*TMEM161B*
91	hsa-miR-203a-3p	*TMEM69*
91	hsa-miR-203a-3p	*TNN*
91	hsa-miR-203a-3p	*TNPO1*
91	hsa-miR-125b-5p	*TOR2A*
91	hsa-miR-34a-5p	*TPCN2*
91	hsa-miR-125b-5p	*TSEN54*
91	hsa-miR-34a-5p	*TSN*
91	hsa-miR-125b-5p	*UBE2R2*
91	hsa-miR-125b-5p	*ULK3*

(*Continued*)

Supplementary Table 2. List of the predicted target genes, associated miRNAs, and prediction scores (score≥90) (*Continued*)

Target Score	miRNA	Gene Symbol
91	hsa-miR-16-5p	*ZC3H13*
91	hsa-miR-34a-5p	*ZDHHC17*
91	hsa-miR-205-5p	*ZEB1*
91	hsa-miR-16-5p	*ZFHX3*
91	hsa-miR-125b-5p	*ZFYVE1*
91	hsa-miR-205-5p	*ZNF652*
91	hsa-miR-205-5p	*ZNF800*
91	hsa-miR-16-5p	*ZNRF3*
90	hsa-miR-205-5p	*ABCD1*
90	hsa-miR-16-5p	*ABHD2*
90	hsa-miR-16-5p	*ADGRL1*
90	hsa-miR-34a-5p	*ADIPOR2*
90	hsa-miR-16-5p	*AK4*
90	hsa-miR-16-5p	*AMOT*
90	hsa-miR-125b-5p	*ANKRD33B*
90	hsa-miR-34a-5p	*ANKRD52*
90	hsa-miR-203a-3p	*ANKS1B*
90	hsa-miR-203a-3p	*ANTXR2*
90	hsa-miR-331-3p	*ARHGEF37*
90	hsa-miR-16-5p	*BAG4*
90	hsa-miR-100-5p	*BAZ2A*
90	hsa-miR-34a-5p	*BCL2L13*
90	hsa-miR-34a-5p	*BRINP1*
90	hsa-miR-16-5p	*C12orf76*
90	hsa-miR-16-5p	*C1orf21*
90	hsa-miR-34a-5p	*CBX3*
90	hsa-miR-125b-5p	*CDC42BPG*
90	hsa-miR-331-3p	*CDC42EP4*
90	hsa-miR-203a-3p	*CDH10*
90	hsa-miR-16-5p	*CDK5R1*
90	hsa-miR-24-3p	*CHD5*
90	hsa-miR-34a-5p	*CLOCK*
90	hsa-miR-203a-3p	*COPS7B*
90	hsa-miR-205-5p	*COX20*
90	hsa-miR-34a-5p	*CPLX2*
90	hsa-miR-125b-5p	*CRB2*
90	hsa-miR-24-3p	*CRIPT*
90	hsa-miR-16-5p	*CSRNP1*
90	hsa-miR-16-5p	*DENND1B*
90	hsa-miR-34a-5p	*DGKZ*
90	hsa-miR-125b-5p	*DRAM2*
90	hsa-miR-125b-5p	*DUSP6*

(*Continued*)

Supplementary Table 2. List of the predicted target genes, associated miRNAs, and prediction scores (score≥90) (*Continued*)

Target Score	miRNA	Gene Symbol
90	hsa-miR-125b-5p	*DYNLT3*
90	hsa-miR-16-5p	*ELL*
90	hsa-miR-16-5p	*ELMSAN1*
90	hsa-miR-125b-5p	*ENPP1*
90	hsa-miR-205-5p	*EPPK1*
90	hsa-miR-125b-5p	*ESRRA*
90	hsa-miR-125b-5p	*EVA1A*
90	hsa-miR-24-3p	*EXOG*
90	hsa-miR-16-5p	*FAM133B*
90	hsa-miR-125b-5p	*FAM169B*
90	hsa-miR-331-3p	*FBLN7*
90	hsa-miR-16-5p	*FERMT2*
90	hsa-miR-125b-5p	*GANC*
90	hsa-miR-203a-3p	*GBE1*
90	hsa-miR-34a-5p	*GMNC*
90	hsa-miR-34a-5p	*GPR158*
90	hsa-miR-125b-5p	*HIF1AN*
90	hsa-miR-203a-3p	*IL24*
90	hsa-miR-16-5p	*ILDR2*
90	hsa-miR-125b-5p	*ITGA8*
90	hsa-miR-16-5p	*JARID2*
90	hsa-miR-205-5p	*KAT2B*
90	hsa-miR-24-3p	*KIF3C*
90	hsa-miR-34a-5p	*KITLG*
90	hsa-miR-203a-3p	*KRT1*
90	hsa-miR-125b-5p	*LFNG*
90	hsa-miR-125b-5p	*LIN28A*
90	hsa-miR-16-5p	*LRIG1*
90	hsa-miR-205-5p	*LYSMD3*
90	hsa-miR-205-5p	*MAGI1*
90	hsa-miR-125b-5p	*MAP3K11*
90	hsa-miR-16-5p	*MAP3K13*
90	hsa-miR-125b-5p	*MAP3K13*
90	hsa-miR-34a-5p	*MBLAC1*

(*Continued*)

Supplementary Table 2. List of the predicted target genes, associated miRNAs, and prediction scores (score≥90) (*Continued*)

Target Score	miRNA	Gene Symbol
90	hsa-miR-125b-5p	*MFHAS1*
90	hsa-miR-125b-5p	*MFSD9*
90	hsa-miR-24-3p	*MICAL2*
90	hsa-miR-34a-5p	*MTMR10*
90	hsa-miR-205-5p	*NACC2*
90	hsa-miR-24-3p	*NAIF1*
90	hsa-miR-24-3p	*NEFM*
90	hsa-miR-24-3p	*NFXL1*
90	hsa-miR-205-5p	*NOTCH2*
90	hsa-miR-16-5p	*NR2C2*
90	hsa-miR-16-5p	*NRN1*
90	hsa-miR-203a-3p	*OPN3*
90	hsa-miR-203a-3p	*PAPSS2*
90	hsa-miR-24-3p	*PDGFRB*
90	hsa-miR-34a-5p	*PDXK*
90	hsa-miR-203a-3p	*PRICKLE2*
90	hsa-miR-34a-5p	*PTGIS*
90	hsa-miR-24-3p	*PURA*
90	hsa-miR-203a-3p	*RAB10*
90	hsa-miR-125b-5p	*RAB3D*
90	hsa-miR-205-5p	*RBM41*
90	hsa-miR-125b-5p	*RETREG2*
90	hsa-miR-34a-5p	*RFX3*
90	hsa-miR-125b-5p	*RHOQ*
90	hsa-miR-205-5p	*RUVBL1*
90	hsa-miR-34a-5p	*SAR1A*
90	hsa-miR-24-3p	*SEC14L5*
90	hsa-miR-203a-3p	*SEC62*
90	hsa-miR-205-5p	*SECISBP2L*
90	hsa-miR-16-5p	*SEMA3A*
90	hsa-miR-24-3p	*SEMA4B*
90	hsa-miR-125b-5p	*SEMA4F*
90	hsa-miR-16-5p	*SEPT2*
90	hsa-miR-34a-5p	*SFT2D1*

(*Continued*)

Supplementary Table 2. List of the predicted target genes, associated miRNAs, and prediction scores (score≥90) (*Continued*)

Target Score	miRNA	Gene Symbol
90	hsa-miR-203a-3p	*SGMS2*
90	hsa-miR-34a-5p	*SHOC2*
90	hsa-miR-16-5p	*SLC36A1*
90	hsa-miR-203a-3p	*SMAD1*
90	hsa-miR-24-3p	*SNCAIP*
90	hsa-miR-203a-3p	*SNTB2*
90	hsa-miR-205-5p	*SORBS1*
90	hsa-miR-24-3p	*SPAG9*
90	hsa-miR-203a-3p	*STRIP2*
90	hsa-miR-16-5p	*SYNRG*
90	hsa-miR-16-5p	*TAB3*
90	hsa-miR-16-5p	*TFCP2L1*
90	hsa-miR-16-5p	*TGFBR3*
90	hsa-miR-125b-5p	*TLE3*
90	hsa-miR-16-5p	*TMC7*
90	hsa-miR-24-3p	*TMEM121B*
90	hsa-miR-34a-5p	*TMEM164*
90	hsa-miR-125b-5p	*TNFSF4*
90	hsa-miR-205-5p	*TP53BP2*
90	hsa-miR-125b-5p	*TRIAP1*
90	hsa-miR-24-3p	*TRIM55*
90	hsa-miR-16-5p	*TRIM66*
90	hsa-miR-16-5p	*UBQLNL*
90	hsa-miR-16-5p	*USP31*
90	hsa-miR-16-5p	*WIPI2*
90	hsa-miR-331-3p	*XPO7*
90	hsa-miR-205-5p	*YAP1*
90	hsa-miR-24-3p	*ZBTB44*
90	hsa-miR-24-3p	*ZCRB1*
90	hsa-miR-24-3p	*ZFR2*
90	hsa-miR-24-3p	*ZNF395*
90	hsa-miR-205-5p	*ZNF655*
90	hsa-miR-125b-5p	*ZNRF3*
90	hsa-miR-100-5p	*ZZEF1*
